# The association between periodontal disease and adverse pregnancy outcomes: a bibliometric analysis from 2000 to 2023

**DOI:** 10.3389/fmed.2025.1526406

**Published:** 2025-01-21

**Authors:** Miaomiao Zhao, Haoxiang Chang, Yuxu Yue, Xinyue Zeng, Shaobang Wu, Xiuyun Ren

**Affiliations:** ^1^Shanxi Medical University School and Hospital of Stomatology, Taiyuan, China; ^2^Shanxi Province Key Laboratory of Oral Diseases Prevention and New Materials, Taiyuan, China

**Keywords:** periodontal disease, adverse pregnancy outcomes, bibliometric analysis, bidirectional, risk factors

## Abstract

**Background:**

Periodontal disease (PD) refers to a chronic inflammatory disorder affecting the supporting tissues of the teeth triggered by bacterial infection and is recognized to promote systemic inflammation, leading to dysfunction in specific organs. Adverse pregnancy outcomes (APOs), including preterm birth, small for gestational age infants, gestational diabetes and preeclampsia, are linked to pregnancy complications. Recently, the correlation between periodontal disease and adverse pregnancy outcomes has garnered global attention. However, bibliometric studies in this area remain limited. This study aimed to visualize knowledge framework and research trends concerning the relationship between periodontal disease and adverse pregnancy outcomes from 2000 to 2023 through bibliometric approaches.

**Methods:**

On September 22, 2024, articles and reviews on the connection between periodontal disease and adverse pregnancy outcomes were retrieved from the Web of Science Core Collection (WOSCC). CiteSpace [6.3.R1 (64-bit) Advanced] was used to perform knowledge mapping and bibliometric studies.

**Results:**

Over the past 23 years, 932 articles from 73 countries were collected, with the U.S. contributing over one-third (355), followed by Brazil (85) and India (59). The literature in this field has experienced multiple growth phases since 2000, with particularly rapid growth observed after 2019. The University of North Carolina (*n* = 34, 3.65%) is the leading institution in terms of publication output, primarily representing the U.S. Notably, the *Journal of Periodontology* and the *American Journal of Obstetrics and Gynecology* are the most frequently cited journals in the fields of periodontology and obstetrics, respectively. These publications are authored by 94 researchers, with Steven Offenbacher being both the most productive and most highly cited author, making significant contributions to the field. A visual analysis of keywords identifies “oral microbiota,” “oral health,” “adverse pregnancy outcomes,” and “global burden” as emerging research hotspots in exploring the correlation between periodontal disease and adverse pregnancy outcomes.

**Conclusions:**

This first bibliometric and visual analysis of periodontal disease and adverse pregnancy outcomes offers a concise overview of the field and suggests future research should focus on risk factors, high-risk populations, oral microbiota, mechanisms, interventions, and international collaboration.

## 1 Introduction

Periodontal disease (PD), which includes plaque-induced gingivitis and periodontitis, is a chronic inflammatory condition affecting the supporting tissues of the teeth. Untreated plaque-induced gingivitis may develop to periodontitis, resulting in tooth mobility and, eventual, the loss of teeth (1, 2). According to reports, the combined prevalence of periodontal disease in adults is 61.6%, with severe periodontitis affecting ~23.6% (3). This not only imposes a significant economic burden but also severely impacts patients' nutrition status and quality of life (4, 5). Moreover, the role of periodontitis in promoting systemic inflammation and leading to dysfunction in specific organs has been widely documented, including associations with cardiovascular diseases, metabolic disorders, neurodegenerative diseases, autoimmune conditions, cancer, and adverse pregnancy outcomes (6–12).

Adverse pregnancy outcomes (APOs) refer to a range of medical events that may negatively impact the health of both mothers and infants during pregnancy, childbirth, or the postpartum period. These include five primary APOs identified from medical birth registries: preterm birth (PB), small for gestational age (SGA), pre-eclampsia (PE), other hypertensive disorders, and gestational diabetes mellitus (GDM), along with other pregnancy-related complications such as miscarriage, stillbirth, placental problems, congenital abnormalities, and postpartum complications (13). According to previous research data, ~30% of women experience at least one unfavorable pregnancy result during their reproductive years (14). The occurrence of APOs is not only a significant contributor to rising rates of morbidity and mortality for mothers and newborns but also crucially elevates the risk of various long-term complications for both, including neurodevelopmental abnormalities, cardiovascular diseases, and other chronic conditions (15).

Since Offenbacher et al. (16) first reported in 1996 that women with periodontitis have nearly seven times the risk of PB and low birth weight (LBW) compared to controls, numerous studies have confirmed the negative consequences of PD on APOs from various perspectives, including epidemiology, shared pathophysiological mechanisms, histological studies, animal experiments, and intervention research. Epidemiological research suggests that the prevalence of PD in pregnant women varies between 20 and 50% (17). Furthermore, multiple studies assessing the correlation between periodontitis and APOs (such as PB, LBW, and PE) have consistently found a positive correlation between the two (18, 19). In 2020, Bobetsis et al. (20) refined the model of the pathogenic mechanisms by which periodontitis affects APOs, along with other relevant research data (21), indicating that periodontitis promotes APOs primarily through three pathways: the uterine oral colonization of periodontal germs, the imbalance of intrauterine inflammatory responses triggered by pro-inflammatory mediators, and the ascent of urogenital pathogens leading to intrauterine infection. Additionally, periodontal pathogens have been found in embryonic tissues by histological investigations, such as the placenta, fetus, and umbilical cord blood, providing strong evidence for the presence of oral infections in the fetal-placental structure (21). Moreover, numerous animal experiments have demonstrated that pregnant mouse models infected with various periodontal pathogens exhibit symptoms such as fetal growth restriction (FGR) and PB, significantly increasing the incidence of LBW in offspring (22–24). These findings collectively suggest a correlation between periodontitis and APOs. However, the effectiveness of clinical interventions for periodontitis in preventing and treating APOs remains unclear. Most studies indicate that periodontal interventions primarily improve periodontal parameters without significantly impacting the occurrence of APOs (25–29). Nonetheless, it is widely recognized that periodontitis is an independent and potential risk factor for APOs.

The aforementioned studies primarily focus on the elevated risk of APOs associated with PD; however, the connection between pregnancy and changes in oral health is bidirectional. Physiological changes during pregnancy significantly affect the immune, respiratory, cardiovascular, and coagulation systems (30). To accommodate the semi-allogeneic fetus, the immune system is suppressed during pregnancy (31). Research indicates that pregnancy impairs neutrophil function, increasing susceptibility to inflammation, which in turn reduces the gingival tissue's ability to resist bacterial-induced inflammatory challenges (32). Additionally, pregnancy induces significant hormonal changes, particularly in sex hormones. Receptors for these hormones are widely expressed in gingival tissues and cells, exacerbating the inflammatory response to plaque-induced periodontal tissue damage (33, 34). Studies have shown that estrogen and progesterone can regulate the microbial composition within periodontal tissues, raising the anaerobic to aerobic bacterial ratio during pregnant. Although a recent cohort analysis revealed no appreciable variations in the percentage of subgingival periodontal pathogens throughout pregnancy, with marked differences observed post-delivery, this still supports the hypothesis that gingival inflammation severity fluctuates during and after pregnancy (17, 35). Moreover, pregnancy hormones may compromise the integrity of periodontal ligament cells, worsening clinical PD symptoms (36). Changes in dietary habits during pregnancy, such as increased refined sugar and protein intake, also promote the growth of plaque-related pathogenic microorganisms (32). These findings suggest that pregnancy-associated physiological traits, hormonal fluctuations, dietary modifications, and reduced oral hygiene interventions contribute to a shift in oral microbial balance, thereby increasing susceptibility to oral diseases.

The complex and bidirectional relationship between pregnancy and PD has emerged as a prominent interdisciplinary topic of interest. However, as knowledge in this area rapidly accumulates, it becomes increasingly challenging to thoroughly understand the knowledge framework and stay updated on the latest research trends, especially for researchers new to the field. Numerous systematic reviews have carried out quantitative assessments on APOs and PD in recent years, concentrating on certain subfields such PD as an APOs risk factor (37), the association between PD and various APOs (38–40), and the effects of non-surgical periodontal treatments on APOs (41). Nevertheless, a comprehensive, macro-level analysis of the knowledge landscape and thematic evolution in this domain remains lacking.

Bibliometrics, first defined by Alan Pritchard in 1969, is a method of quantitative analysis that examines relevant literature, coverage areas, and citation patterns to reveal and evaluate development trends, author collaboration networks, and the transfer of knowledge within a particular research domain (42, 43). It has been used extensively in medical research over the past few decades, including studies on PD (44), obstetric and gynecological disorders (45), cardiovascular diseases (46), and metabolic diseases (47). In recent years, the quantity of articles about PD and APOs has grown rapidly, yet systematic analyses using bibliometric methods remain scarce. Therefore, this study attempts to outline the general framework of research on the association between PD and APOs by summarizing and analyzing the quantitative data and research structure currently available in this subject using a bibliometric approach. The findings highlight the research priorities and emerging trends, providing scientific evidence for future research directions and academic decision-making.

## 2 Methods

### 2.1 Data source and search strategy

The Web of Science Core Collection (WOSCC) database (https://www.webofscience.com) served as the data source for this investigation, which widely regarded as the premier database for bibliometric analysis (48). The search query employed was “TS = (“periodontal disease” OR periodont^*^ OR gingiv^*^ OR “tooth loss” OR “tooth migration” OR “tooth mobility”) AND TS = (“Adverse pregnancy outcomes” OR “Miscarriage” OR “Preterm birth” OR “Low birth weight” OR “Congenital anomalies” OR “Stillbirth” OR “Maternal complications” OR “Placental issues” OR “Postpartum complications”).” All retrieved documents were independently evaluated by two reviewers (MMZ and HXC) based on titles, abstracts, and keywords to exclude studies unrelated to PD and APOs. In cases of disagreement between the two reviewers, the judgment of a third reviewer (YXY) was deemed final. To minimize bias from database updates, the literature search was completed in a single day (2024-09-22).

### 2.2 Inclusion criteria

Publications that meet all of the following criteria are included in the subsequent analysis, while others are excluded:

(1) Articles or review papers related to the field of PD and APOs;
(2) Articles or review papers published between 2000 and 2023;
(3) Articles or review papers written in English.

### 2.3 Exclusion criteria

Publications meeting any of the following criteria are excluded from this study:

(1) Publications before 2000 or after 2023;
(2) Publication types other than articles, review papers, and early access;
(3) Non-English publications.

### 2.4 Data collection and data analysis

The collected papers were exported as “Full Record and Cited References” and saved in “Plain Text” format, with the files named “download_.txt.” We utilized Microsoft Office Excel 2019 to manage data and analyze annual publications. Furthermore, CiteSpace [6.3.R1 (64-bit) Advanced] was employed to analyze the data and create visual representations of scientific knowledge maps.

The Java-based citation visualization program CiteSpace, created by Chaomei Chen's group, gives researchers an experimental platform to investigate new concepts and assess current methodologies. It allows for in-depth analysis of literature from various perspectives, identifying research trends and hotspots in particular domains, and presenting them visually. The generated knowledge maps offer researchers an intuitive approach to comprehending the development of research focuses, enabling them to predict future trends in areas of interest (49).

## 3 Results

### 3.1 General information

**Figure 1:**
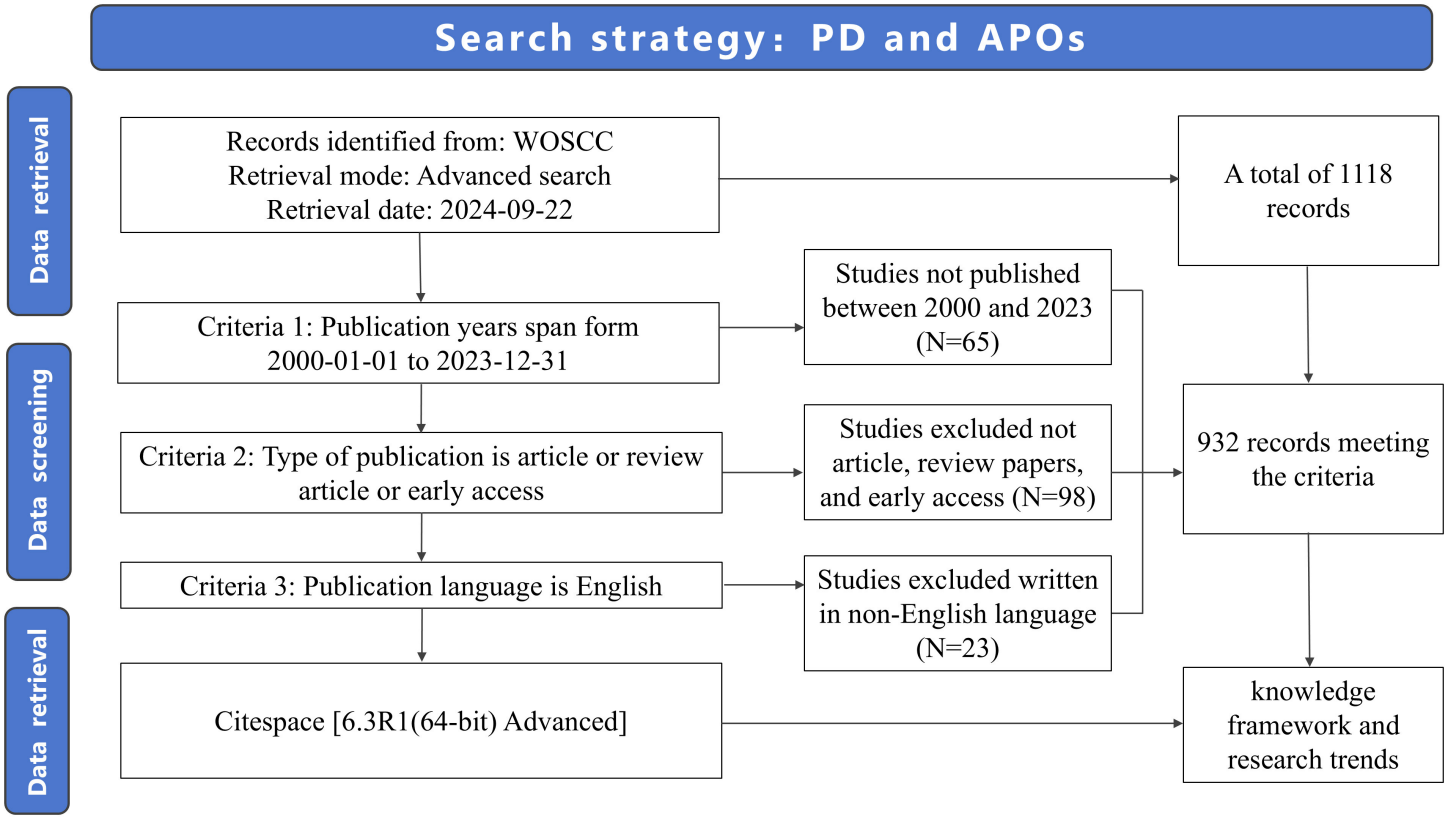
Flowchart of the search strategy.

**Figure 2:**
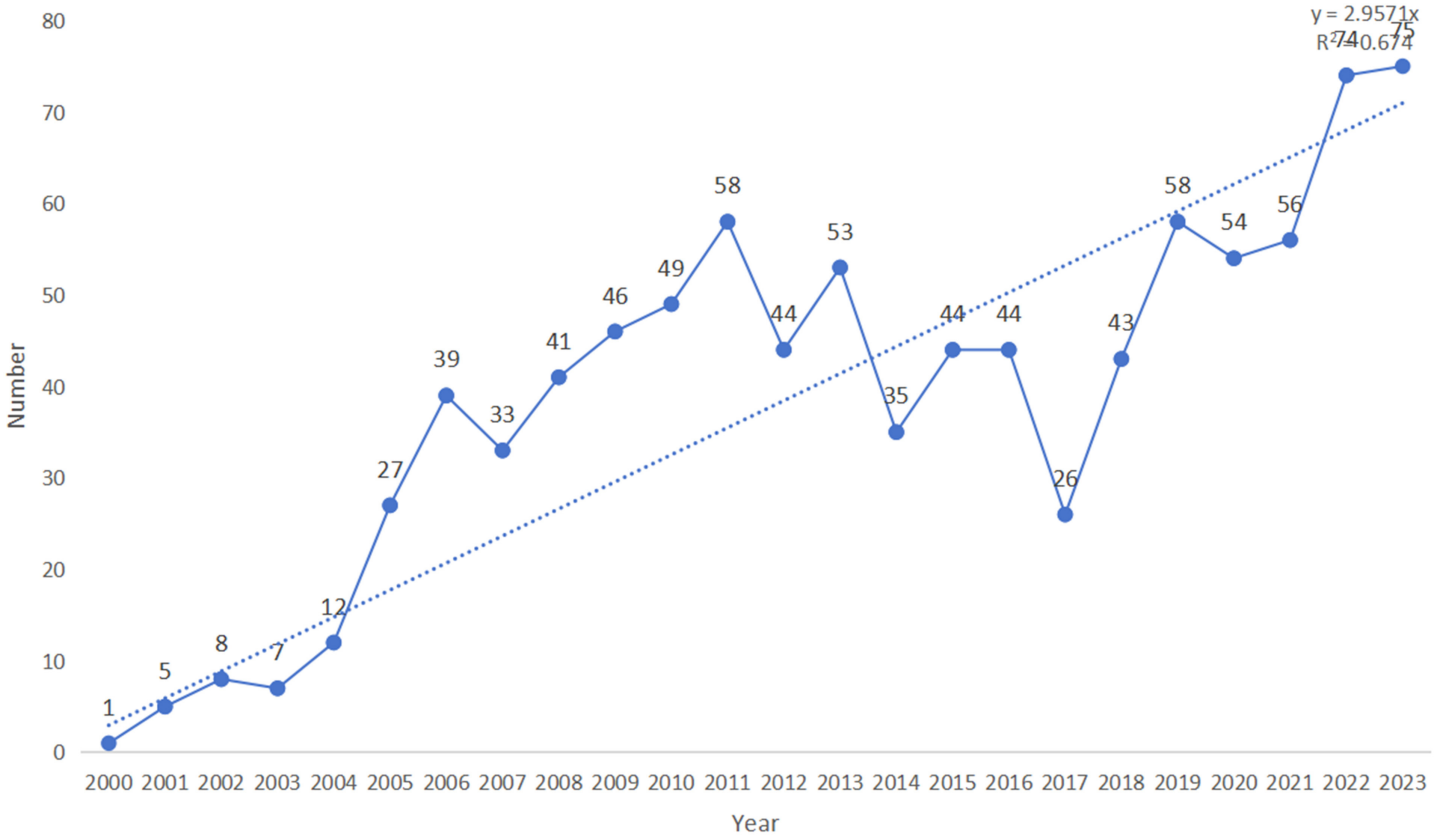
The number of published studies over time.

In total, 1,118 publications were obtained as a result of the literature search. After screening, 932 publications were included in the subsequent bibliometric analysis, comprising 728 research articles (728/932, 78.112%) and 208 review articles (208/932, 22.318%). Of the four early access articles, three were categorized as research articles, and one as a review article (Figure 1; Supplementary Table 1). From 2000 to 2003, the annual quantity of publications in this area remained below ten. However, since 2004, the annual publication count has significantly increased (R^2^ = 0.674), peaking at 75 publications in 2023 (Figure 2; Supplementary Table 2). As illustrated in Figure 2, the number of publications steadily grew between 2004 and 2011, reaching 58 publications in 2011. This growth indicates the increasing academic attention toward this research area. During the period from 2012 to 2018, publication volume fluctuated but remained at a relatively high level (averaging more than 40 publications per year). Between 2019 and 2023, the publication volume experienced explosive growth, culminating in the peak of 75 publications in 2023. Since 2019, the number of publications has remained consistently high, reflecting active research activities and significant interest from the academic community. This trend indicates that academic interest in the relationship between PD and APOs has steadily increased since 2004, with research activities remaining robust from 2007 to 2023, highlighting sustained attention from the international academic community.

Overall, since 2000, this research field has undergone several phases of growth, particularly with the rapid expansion observed after 2019. This trend reflects the increasing attention within the academic community, suggesting that more research outcomes are likely to be published in the future, driving further advancements in this field.

### 3.2 Cooperation network analysis

#### 3.2.1 Distribution of countries/regions

**Figure 3:**
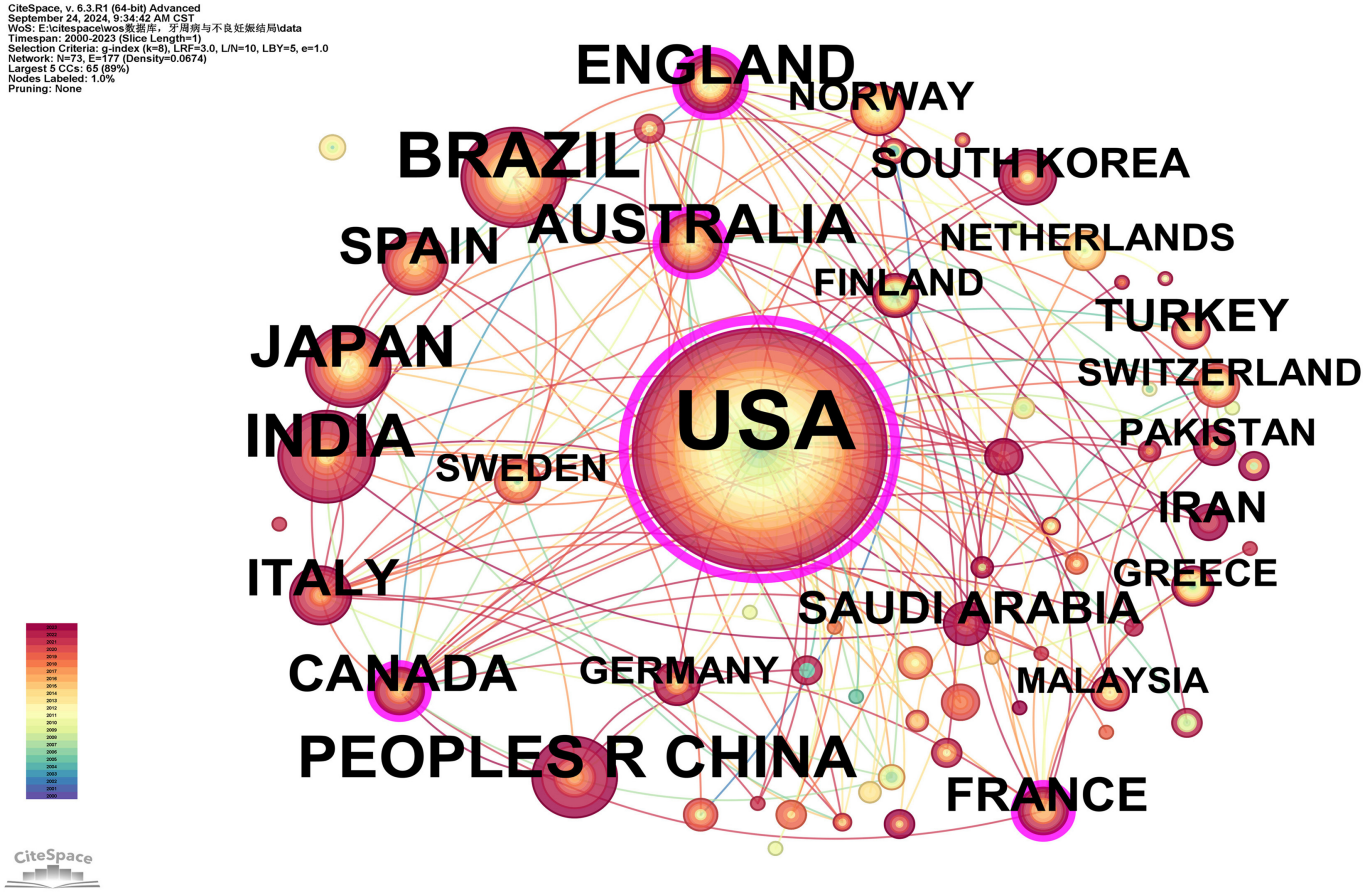
Cooperation network map of countries/regions. After standardizing the sample literature (932 publications), the data were imported into the software CiteSpace for analysis of the selected database, with the time period set from 2000 to 2023. The analysis item was set to “Country,” and a co-occurrence map of countries corresponding to studies on the relationship between PD and APOs was generated. The size of the nodes represents the number of publications, and the connections between nodes represent collaborations. The different colors represent the years of collaboration. The outermost purple rings indicate the levels of betweenness centrality (BC), with nodes of high BC considered as key points in the research field [g-index (*k* = 8), *N* = 73 (number of network nodes), *E* = 117 (number of connections), Density = 0.0674 (network density)].

A total of 932 publications originated from 73 countries, with the United States contributing over one-third of the literature (355/932, 38.09%), followed by Brazil (85 publications) and India (59 publications; see Supplementary Table 3). This highlights the significant influence of the U.S. in this field. Furthermore, the U.S. exhibited the highest betweenness centrality (BC = 0.77), indicating a high level of collaboration with other countries, followed by Australia (0.14) and Canada (0.13; see Supplementary Table 3). The visualization of country/region collaborations reveals that the U.S.'s closest collaborators are primarily developed nations, including Australia, Canada, France, Germany, Italy, and Sweden (Figure 3).

#### 3.2.2 Distribution of institutions

**Figure 4:**
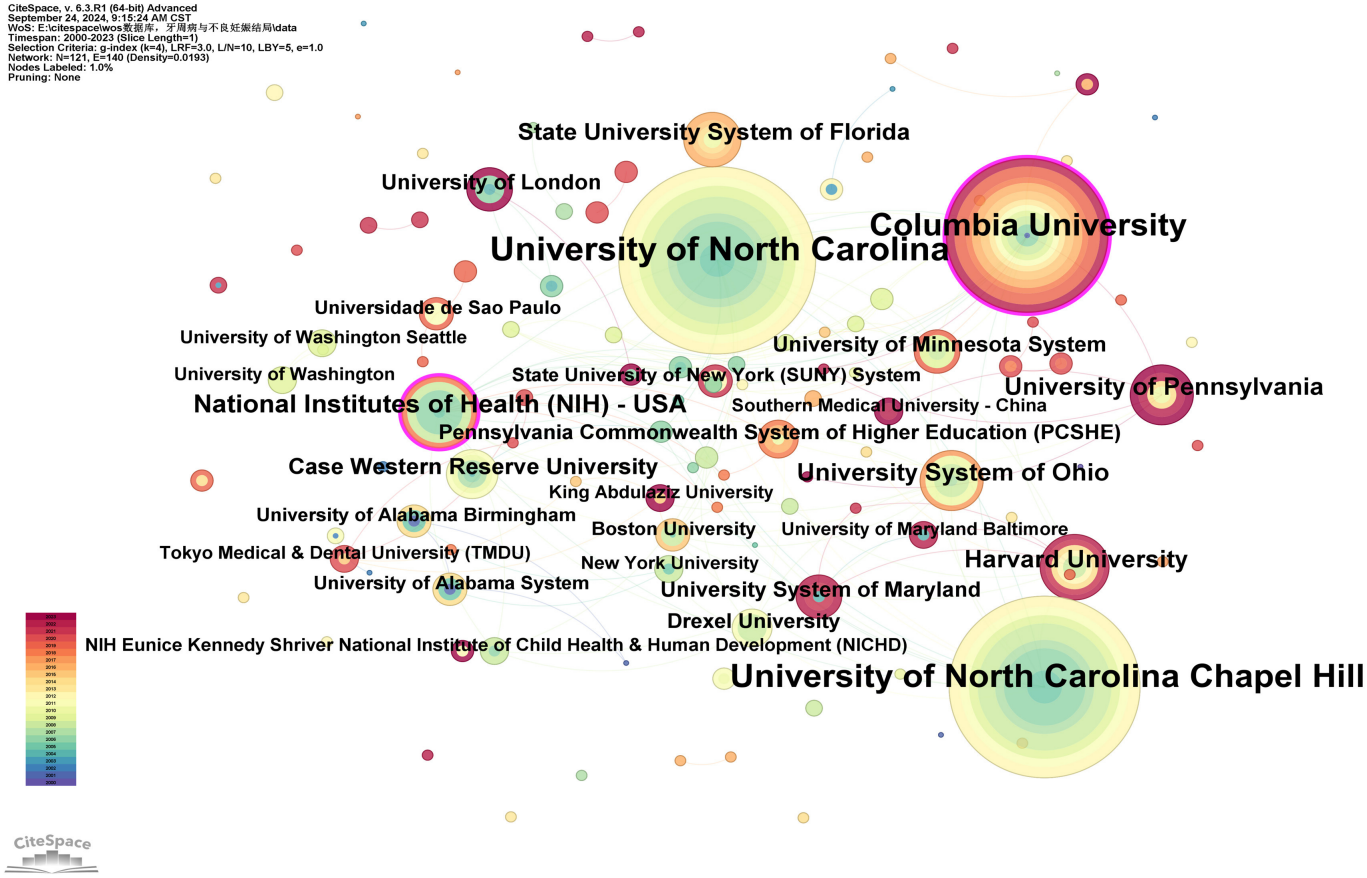
Cooperation network map of institutions. After normalizing the sample literature (932 publications), the data was imported into CiteSpace for analysis. The selected time period spanned from 2000 to 2023, with the analysis focused on the “institution” level to construct a co-occurrence network map of institutions researching the relationship between PD and APOs. Node size represents the number of publications, while the links between nodes indicate collaborative efforts. Different colors represent the years of cooperation. The outermost purple ring denotes the level of centrality, with high centrality nodes considered key points in the research field. The network parameters are as follows: g-index (*k* = 4), *N* = 121 (number of network nodes), *E* = 140 (number of connections), and Density = 0.0193 (network density).

Researchers from 121 different institutions have made contributions to the study of this field. The top three institutions by number of publications are the University of North Carolina, the University of North Carolina at Chapel Hill, and Columbia University, with 34, 33, and 28 publications, respectively. Two institutions have a betweenness centrality (BC) >0.1: Columbia University (0.16) and the National Institutes of Health (NIH)—USA (0.11; see Supplementary Table 4). Figure 4 reveals the following characteristics of international research institutions and their collaborations in the study of the relationship between PD and APOs:

(I) Key institutions: in terms of publication volume, several institutions have emerged as key players in research on the relationship between PD and APOs. The largest node, representing the institution with the highest number of publications, is the University of North Carolina, followed by the University of North Carolina at Chapel Hill, Columbia University, the National Institutes of Health (NIH)—USA, Harvard University, the University of Pennsylvania, the University System of Ohio, the State University System of Florida, Case Western Reserve University, and the University of London. These institutions form the core of research on the PD-APOs relationship.

(II) Research Trends and Geographic Distribution: In terms of publication volume, 17 out of the top 20 institutions are located in the United States, highlighting the significant influence of American institutions in this field. This aligns with the findings in Figure 3. The geographic distribution of research institutions is relatively widespread, with high levels of interest in this research area observed globally. Notably, research activity is particularly prominent in North Carolina, New York, Massachusetts, Ohio, Maryland, and Pennsylvania. Additionally, countries such as Brazil, India, Japan, China, Australia, and the United Kingdom also show high levels of research activity. This global distribution underscores the importance of PD-APOs relationship research and its broad international attention.

#### 3.2.3 Authors and co-cited authors

**Figure 5:**
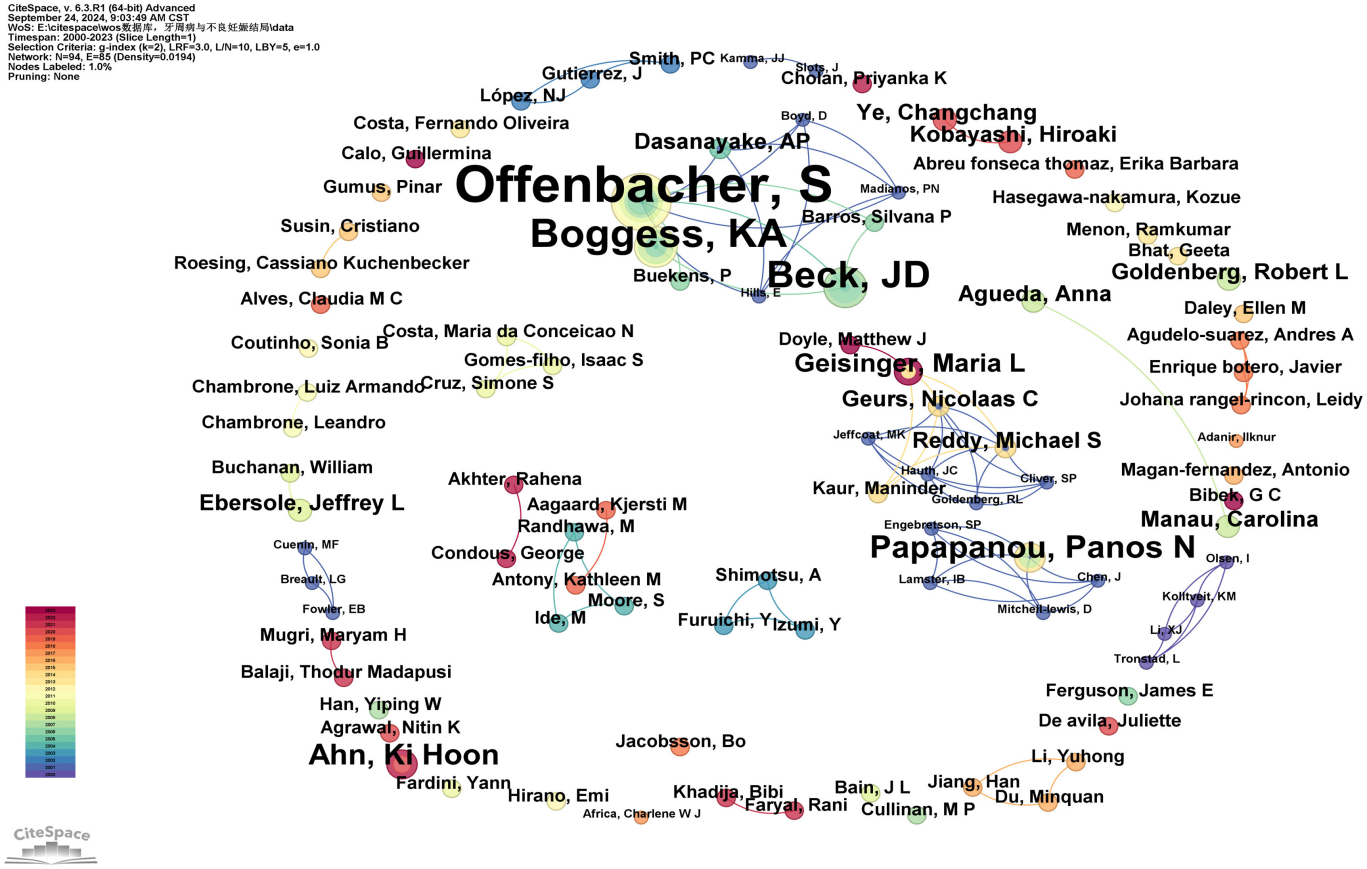
Cooperation network map of authors. After normalizing the sample literature (932 publications), the data was imported into CiteSpace for analysis. The selected time period spanned from 2000 to 2023, with the analysis focused on the “author” level to construct a co-occurrence network map of authors researching the relationship between PD and APOs. Node size represents the number of publications by each author, while the links between nodes indicate collaborative efforts. Different colors represent the years of cooperation. The network parameters are as follows: g-index (*k* = 2), *N* = 94 (number of network nodes), *E* = 85 (number of connections), and Density = 0.0194 (network density).

In total, 94 authors have been involved in contributing to the research publications on the relationship between PD and APOs. Only five authors have published more than five articles: Steven Offenbacher (24 articles), Boggess, KA (11 articles), Beck, JD (9 articles), Papapanou, Panos N (6 articles), and Ahn, Ki Hoon (5 articles; see Supplementary Table 5). As shown in Figure 5, the number of connections is fewer than the number of nodes, indicating a low network density, which suggests limited collaboration among key researchers in this field. Most researchers work independently with minimal co-authorship. Notably, some influential authors, especially Steven Offenbacher, Boggess, KA, and Beck, JD, have formed a significant research network in this field. Steven Offenbacher, in particular, has been an active contributor since publishing his first paper in 2001 and continued through 2023. Additionally, new authors contribute their first papers annually, indicating sustained interest and vitality in this research area (see Supplementary Table 5).

**Figure 6:**
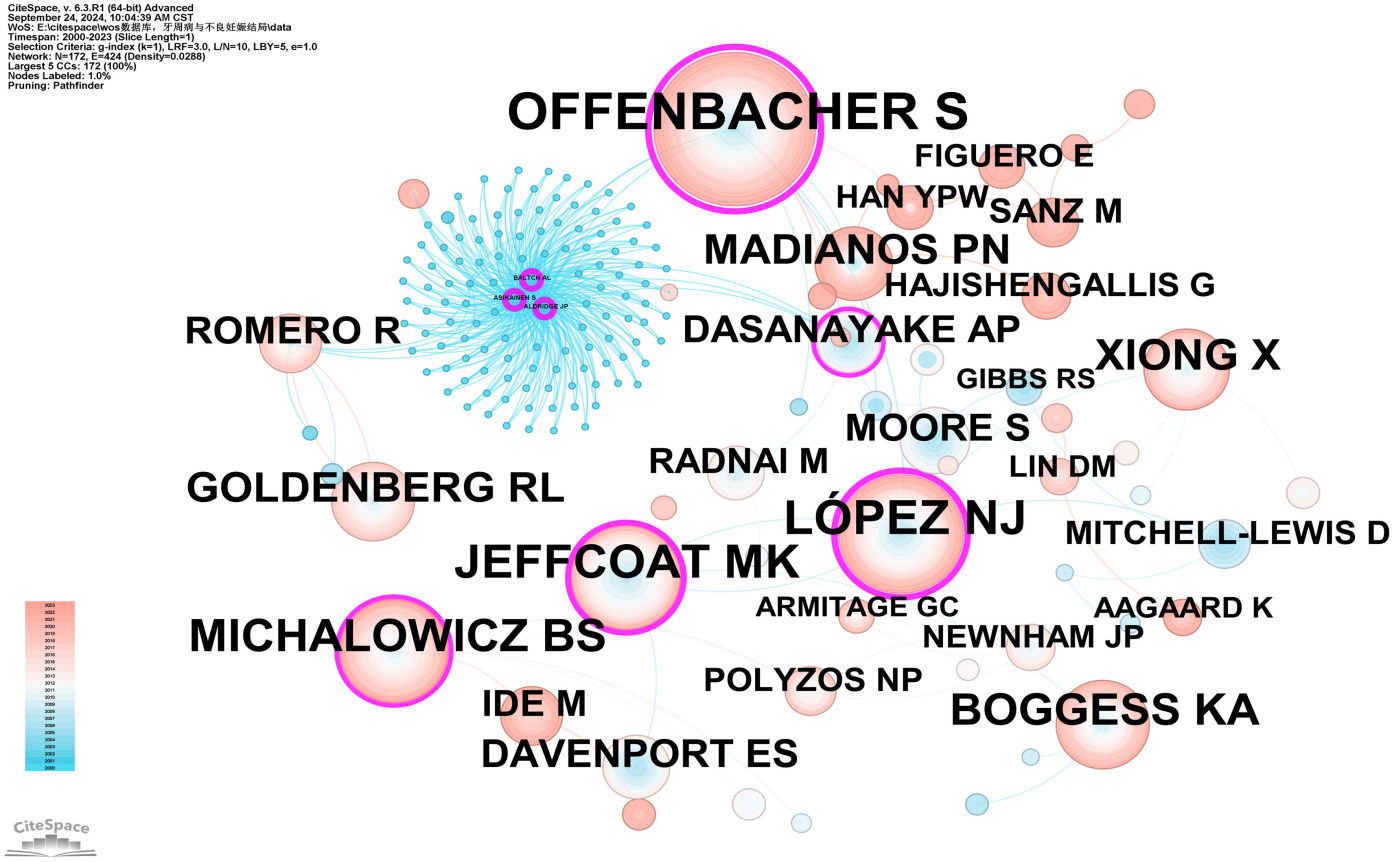
Co-citation network map of authors. After normalizing the sample literature (932 publications), the data was imported into CiteSpace for analysis. The selected time period spanned from 2000 to 2023, and the analysis focused on the “Author Co-citation Network” to construct a co-citation network map of authors researching the relationship between PD and APOs. Node size represents the number of citations each author received, while the links between nodes indicate co-citations within the same paper. Different colors represent the years in which the citations occurred. The network parameters are as follows: g-index (*k* = 1), *N* = 172 (number of network nodes), *E* = 424 (number of connections), and Density = 0.0288 (network density).

Co-citation refers to the situation where two or more authors are cited together in the same publication or across multiple publications. In our studies, all of the top ten co-cited authors have been cited over 100 times. The most frequently cited co-cited author is Steven Offenbacher (575 citations), followed by LÓPEZ NJ (345 citations), JEFFCOAT MK (310 citations), MICHALOWICZ BS (270 citations), and BOGGESS KA (224 citations). Additionally, eight authors have a centrality value exceeding 0.10 (see Supplementary Table 6), with Steven Offenbacher having the highest betweenness centrality (BC) value of 0.74, followed by ALDRIDGE JP (0.60), ASIKAINEN S (0.60), BALTCH AL (0.60), LÓPEZ NJ (0.47), JEFFCOAT MK (0.29), MICHALOWICZ BS (0.16), and DASANAYAKE AP (0.10). As illustrated in Figure 6, purple circles represent these highly central co-cited writers, highlighting their significant bridging role within the research network.

### 3.3 Co-cited document and journals

**Figure 7:**
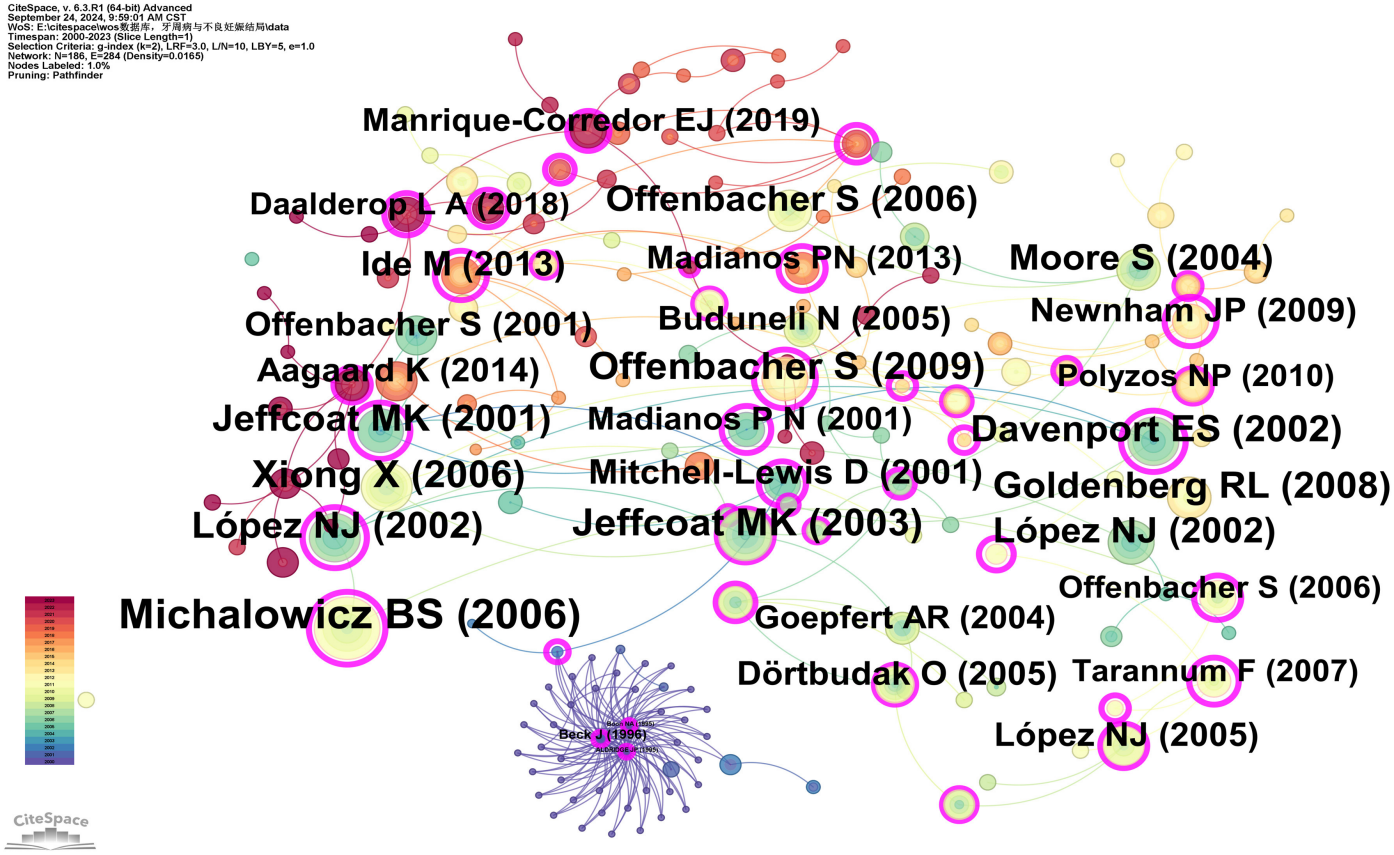
Co-cited network map of documents. After normalizing the sample literature (932 publications), the data were imported into CiteSpace for analysis. The selected time period spanned from 2000 to 2023, with the analysis focusing on “documents” to construct a co-citation network map of literature related to the relationship between PD and APOs. Node size represents the number of citations each document received, while links between nodes indicate that the documents were co-cited in the same paper. The different colors indicate the years in which citations occurred. The outermost purple ring signifies the degree of centrality, with nodes of high centrality regarded as key elements in the research field. The network parameters are as follows: g-index (*k* = 2), *N* = 186 (number of network nodes), E = 284 (number of connections), and Density = 0.0165 (network density).

Co-citation analysis identifies core literature and research hotspots within a specific field by recognizing frequently co-cited publications. Using CiteSpace software with the g-index set to 2, a total of 186 co-cited publications were filtered. The three most cited publications are: Michalowicz BS, 2006, *The New England Journal of Medicine* (96 citations); Jeffcoat MK, 2003, *Journal of Periodontology* (64 citations); and Xiong X, 2006, *BJOG: An International Journal of Obstetrics and Gynecology* (63 citations). Additionally, three articles have a betweenness centrality (BC) of 1.00 or higher: Newnham JP, 2009, *Obstetrics and Gynecology* (BC = 1.09); Tarannum F, 2007, *Journal of Periodontology* (BC = 1.01); and Offenbacher S, 2009, *Obstetrics and Gynecology* (BC = 1; see Supplementary Table 7). Michalowicz BS, 2006, *The New England Journal of Medicine* (96 citations) and Newnham JP, 2009, *Obstetrics and Gynecology* (BC = 1.09) represent the most cited and the highest centrality articles, respectively, indicating their broad recognition and influence among scholars in the field (Figure 7).

**Figure 8:**
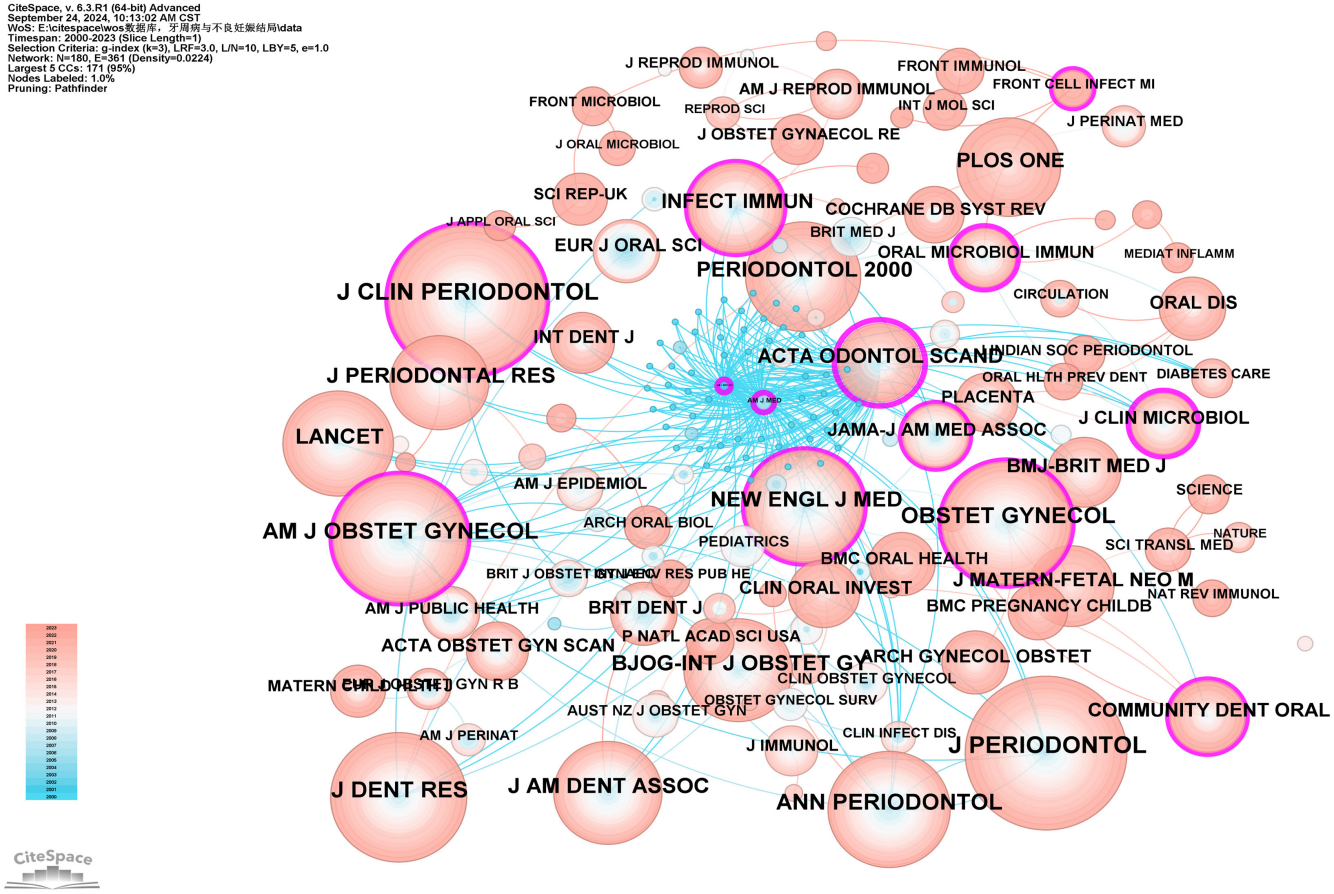
Co-cited network map of journals. After normalizing the sample literature (932 publications), the data were imported into CiteSpace for analysis. The selected time period spanned from 2000 to 2023, with the analysis focusing on “journals” to construct a co-citation network map of journals related to the relationship between PD and APOs. Node size represents the number of citations each journal received, while links between nodes indicate co-citation in the same paper. The different colors indicate the years in which citations occurred. The outermost purple ring signifies the degree of centrality, with nodes of high centrality regarded as key elements in the research field. The network parameters are as follows: g-index (*k* = 3), *N* = 180 (number of network nodes), *E* = 361 (number of connections), and Density = 0.0224 (network density).

Co-citation journal analysis identifies core journals and academic communication channels within a specific research area by analyzing the frequency of co-citation among journals. Using CiteSpace software with the g-index set to 3, a total of 180 journals were identified. As illustrated in Figure 8, the most cited journal and the journal with the highest betweenness centrality (BC) are the *Journal of Periodontology* (792 citations) and *Acta Odontologica Scandinavica* (BC = 0.55), respectively. Based on their citation frequency and related research areas, these journals are classified as core journals in periodontal research and obstetrics and gynecology research. The top five core journals in periodontal research based on citation frequency are: *Journal of Periodontology* (792 citations), *Journal of Clinical Periodontology* (683 citations), *Journal of Dental Research* (592 citations), *Annals of Periodontology* (507 citations), and *Periodontology 2000* (414 citations). The top five core journals in obstetrics and gynecology research based on citation frequency are: *American Journal of Obstetrics and Gynecology* (544 citations), *Obstetrics and Gynecology* (494 citations), *BJOG: An International Journal of Obstetrics & Gynecology* (352 citations), *Archives of Gynecology and Obstetrics* (138 citations), and *Acta Obstetricia et Gynecologica Scandinavica* (131 citations). The high citation rates of these journals indicate their significant influence within their respective fields (see Supplementary Table 8).

### 3.4 Keyword analysis

#### 3.4.1 Keyword co-occurrence

**Figure 9:**
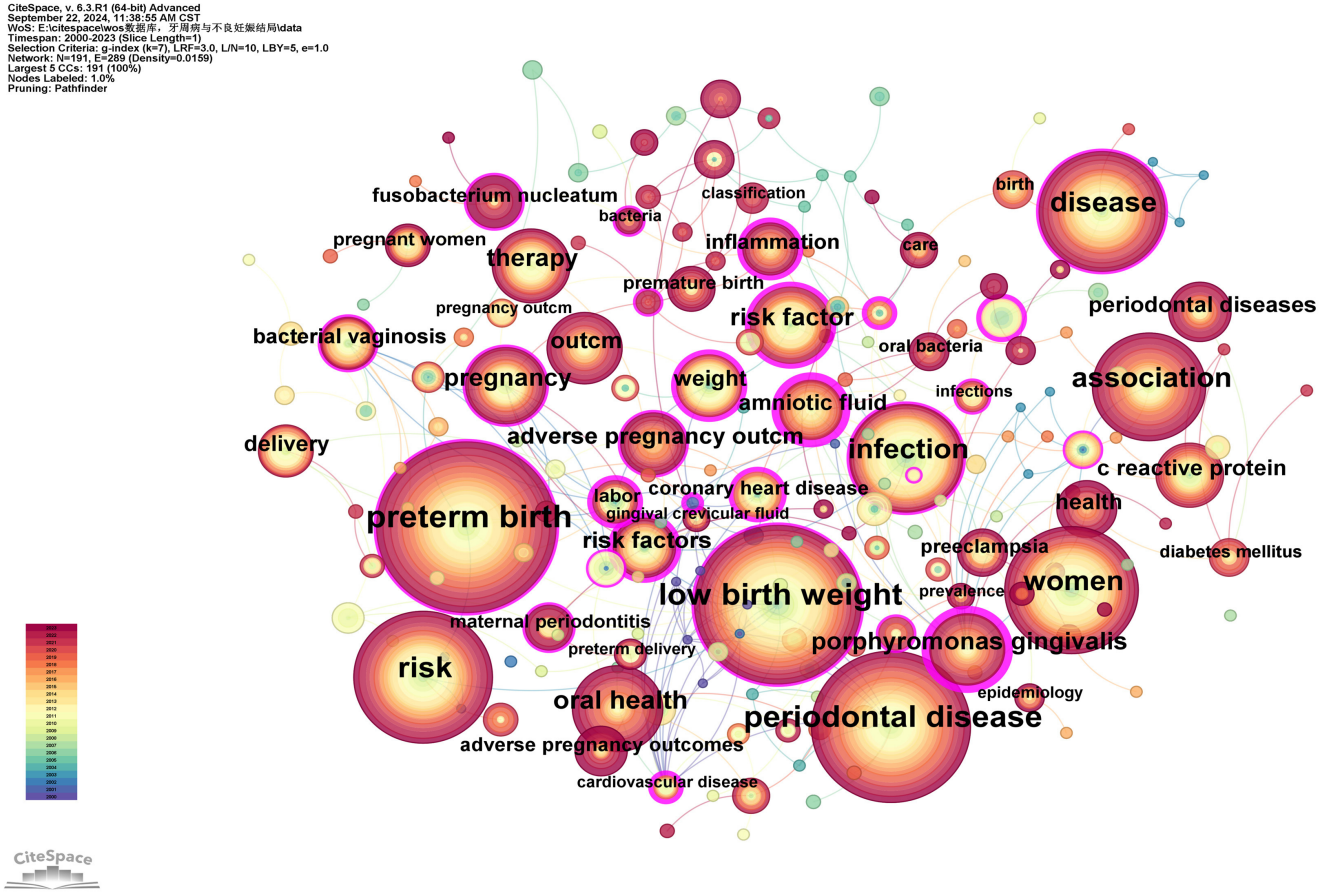
Keywords co-occurrence network map. After normalizing the sample literature (932 publications), the data was imported into CiteSpace for analysis. The selected time period spanned from 2000 to 2023, focusing on the “keywords” level to construct a co-occurrence network map of keywords related to the relationship between PD and APOs. Node size represents the frequency of keyword occurrences, while links between nodes indicate co-occurrence between different keywords. The years of occurrence are represented by different colors. The degree of centrality is shown by the outermost purple ring; nodes with high centrality are regarded as important locations in the research field. The network parameters are as follows: g-index (*k* = 7), *N* = 191 (number of network nodes), *E* = 289 (number of connections), and Density = 0.0159 (network density).

Keywords serve as concise summaries of articles and analyzing them helps identify research hotspots and emerging trends. This study identified a total of 191 keywords, with the top 10 most frequently occurring keywords being: “preterm birth” (*n* = 442), “low birth weight” (*n* = 371), “periodontal disease” (*n* = 350), “risk” (*n* = 274), “women” (*n* = 243), “disease” (*n* = 214), “infection” (*n* = 202), “association” (*n* = 195), “oral health” (*n* = 128), and “pregnancy” (*n* = 108; see Supplementary Table 9). Betweenness centrality (BC) values are used to measure a node's bridging role in the network, indicating its ability to connect other nodes. Keywords with high BC values typically represent potentially influential research directions or interdisciplinary research points. Using a threshold of BC = 0.2, the keywords are ranked from high to low as follows: “amniotic fluid” (0.45), “*Porphyromonas gingivalis*” (0.44), “risk factor” (0.39), “coronary heart disease” (0.36), “risk factors” (0.32), “labor” (0.31), “cardiovascular disease” (0.31), “inflammation” (0.30), “united states” (0.25), “lipopolysaccharide” (0.24), “weight” (0.22), “prematurity” (0.22), and “bacterial vaginosis” (0.20; see Supplementary Table 9; Figure 9).

#### 3.4.2 Keyword clustering

**Figure 10:**
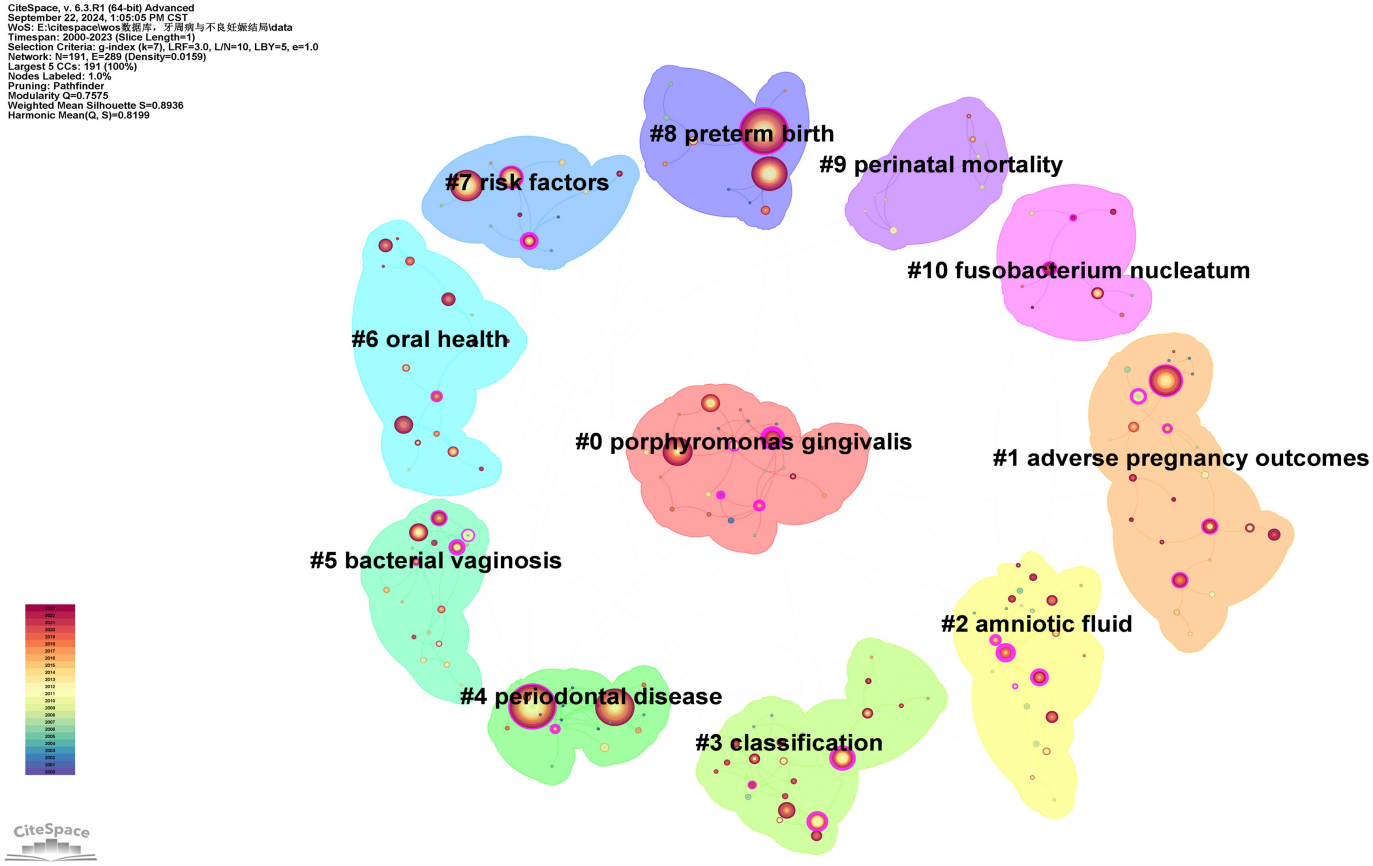
Keywords cluster network map.

Using the extracted keywords for cluster analysis, a total of 11 distinct clusters with different colors were generated, as shown in Figure 10. The average silhouette and modularity score of the clusters are 0.8936 and 0.7575, correspondingly, indicating effective network segmentation. The clusters exhibit clear structures, high quality, and are well-separated. Furthermore, the clusters are labeled in the following order: “*Porphyromonas gingivalis* (#0),” “adverse pregnancy outcomes (#1),” “amniotic fluid (#2),” “classification (#3),” “periodontal disease (#4),” “bacterial vaginosis (#5),” “oral health (#6),” “risk factors (#7),” “preterm birth (#8),” “perinatal mortality (#9),” and “*Fusobacterium nucleatum* (#10).” The clusters with the most keywords are “*Porphyromonas gingivalis* (#0)” and “adverse pregnancy outcomes (#1),” each containing 24 keywords (see Supplementary Table 10). The larger cluster sizes may indicate that these two areas have a broader research foundation compared to other clusters. The cluster with the highest silhouette score is “periodontal disease (#4),” which is commonly used to evaluate cluster cohesion and separation. A higher silhouette score suggests a high similarity of keywords within the cluster and a low similarity with other clusters. Below are some keywords and relevant descriptions for each cluster:

Cluster #0 includes keywords such as “*Porphyromonas gingivalis*,” “coronary heart disease,” “C-reactive protein,” “bone mineral density,” and “atherosclerosis.” *Porphyromonas gingivalis* is a primary pathogen linked to PD, often causing chronic inflammation and destruction of periodontal tissues (50). This cluster is the largest in the Figure 10, indicating that a significant amount of research focuses on the pathogenic mechanisms of this bacterium in relation to PD.

Cluster #1 includes keywords such as “adverse pregnancy outcomes,” “pregnancy outcome,” “periodontitis,” and “maternal periodontitis.” This cluster directly addresses the relationship between PD and APOS, including PB, LBW, and FGR (7).

Cluster #2 includes keywords such as “amniotic fluid,” “oral bacteria,” “periodontal pathogens,” “oral health,” and “bone loss.” This cluster emphasizes the role of amniotic fluid infections or inflammation in pregnancy complications, particularly in relation to the potential connection with PD. Bacteremia caused by PD may lead to the invasion of bacteria or inflammatory mediators into the amniotic fluid, resulting in chorioamnionitis or other membrane infections, thereby increasing the risk of PB (51, 52). This cluster discusses how infections induced by periodontal disease can be detected through amniotic fluid analysis.

Cluster #3 includes keywords such as “classification,” “peri-implant diseases,” “risk factor,” “gestational diabetes mellitus,” and “periodontal medicine.” This cluster pertains to the classification studies of PD, which may include various subtypes of periodontitis and their differing impacts on systemic health (53). It likely addresses the pathogenesis of different types of PD and their correlation with pregnancy outcomes. Taxonomic studies can enhance the understanding of the etiology of PD and facilitate the development of personalized treatment plans targeting different types of periodontal conditions.

Cluster #4 includes keywords such as “periodontal disease,” “low birth weight,” “cardiovascular disease,” “periodontal diseases,” and “bacteria.” This cluster centers around PD, which represents a core topic within the research field. It addresses the pathophysiology of PD, the types of pathogenic bacteria involved, and how oral health management can prevent complications during pregnancy. Such studies assist in elucidating the direct relationship between PD and APOs.

Cluster #5 includes keywords such as “bacterial vaginosis,” “adverse pregnancy outcomes,” “delivery,” “randomized controlled trials,” and “prenatal care.” Bacterial vaginosis is a prevalent infection during pregnancy, often linked to PB and various other pregnancy-related complications (54, 55). This cluster explores the similarities or common mechanisms between bacterial vaginosis and PD, potentially focusing on how dysbiosis can lead to systemic inflammation, thereby affecting pregnancy outcomes.

Cluster #6 includes keywords such as “oral health,” “diabetes mellitus,” “periodontal diseases,” “periodontal disease,” and “dental care.” Oral health plays an important part as a significant factor influencing systemic health in this cluster. The relationship between periodontal health and pregnancy health is an important topic. This cluster investigates how oral hygiene management and periodontal treatment can improve pregnancy outcomes, particularly by reducing the risks of PB and LBW.

Cluster #7 includes keywords such as “risk factors,” “periodontal disease,” “adverse effects,” “periodontal diseases,” “complications,” “regression analysis,” and “possible association.” This cluster discusses various risk factors associated with PD and APOs.

Cluster #8 includes keywords such as “preterm birth,” “risk,” “*Porphyromonas gingivalis*,” “maternal oral health,” and “nursing.” PB is a core topic in the study of PD and pregnancy outcomes. This cluster discusses how periodontal infections may increase the risk of PB through systemic inflammatory responses or bacterial translocation (21). Such studies focus on how periodontal treatment interventions can reduce the rate of PB.

Cluster #9 includes keywords such as “perinatal mortality,” “randomized controlled trial,” “respiratory distress syndrome,” “calcium supplementation,” and “human chorionic gonadotropin.” This cluster investigates the potential link between perinatal mortality and PD. It discusses whether periodontal infections affect fetal survival rates, particularly in high-risk pregnancy populations, and whether PD significantly increases the risk of perinatal mortality as a complication (56).

Cluster #10 includes keywords such as “*Fusobacterium nucleatum*,” “oral microbiome,” “pregnant women,” “fusobacterium,” and “type I signal peptidase.” *Fusobacterium nucleatum* is another pathogen closely associated with PD. Additionally, this bacterium has garnered attention for its pathogenicity during pregnancy. This cluster explores how this bacterium may enter the placenta or fetus through bloodstream dissemination, potentially leading to APOs (57).

#### 3.4.3 Keyword timeline

**Figure 11:**
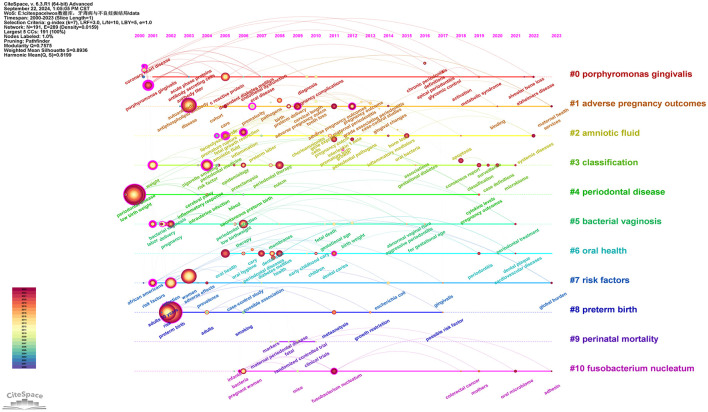
Keywords timeline network map. This figure illustrates the evolutionary trends of keywords related to PD and APOs from 2000 to 2023. In the figure, nodes represent keywords, with the size of each node indicating the frequency of the keyword, and the color representing the time of the keyword's first appearance. Links between nodes indicate co-occurrence relationships between the keywords.

After standardizing the sample literature (932 publications), the data was imported into CiteSpace for analysis of the chosen database, spanning from 2000 to 2023, with each time slice set to 1 year. The analysis item was set to “keywords,” resulting in a keyword co-occurrence map. By utilizing the timeline features, a timeline view of the role of PD in APOs from 2000 to 2023 was generated. As shown in Figure 11 and Supplementary Table 11, “periodontal disease” and “adverse pregnancy outcomes” have consistently been prominent keywords from 2000 to 2023, with a significant increase in research attention particularly between 2010 and 2023, highlighting the growing relevance and continuity of these topics. “*Porphyromonas gingivalis* (#0)” represents the largest cluster, and its prominence has notably increased since 2010, indicating its recognition as a key pathogen in the study of PD and APOs. Additionally, the timeline graph reveals connections between various keywords, such as the strong associations of “risk factor(s)” with “preterm birth,” “periodontitis,” “oral health,” and “cigarette smoking.” As time progresses, these intersections become more frequent, demonstrating an increasing convergence of research topics. Moreover, the crossing and connections of lines in the timeline illustrate the overlap of research fields in specific years. For example, “bacterial vaginosis” was a major topic of discussion from 2011 to 2015, during which “*Fusobacterium nucleatum*” also emerged and intersected with “oral microbiome,” a term that appeared after 2021. This reflects the growing body of research that bridges oral health with systemic health, offering more opportunities for interdisciplinary research in the future.

#### 3.4.4 Keyword time zone

**Figure 12:**
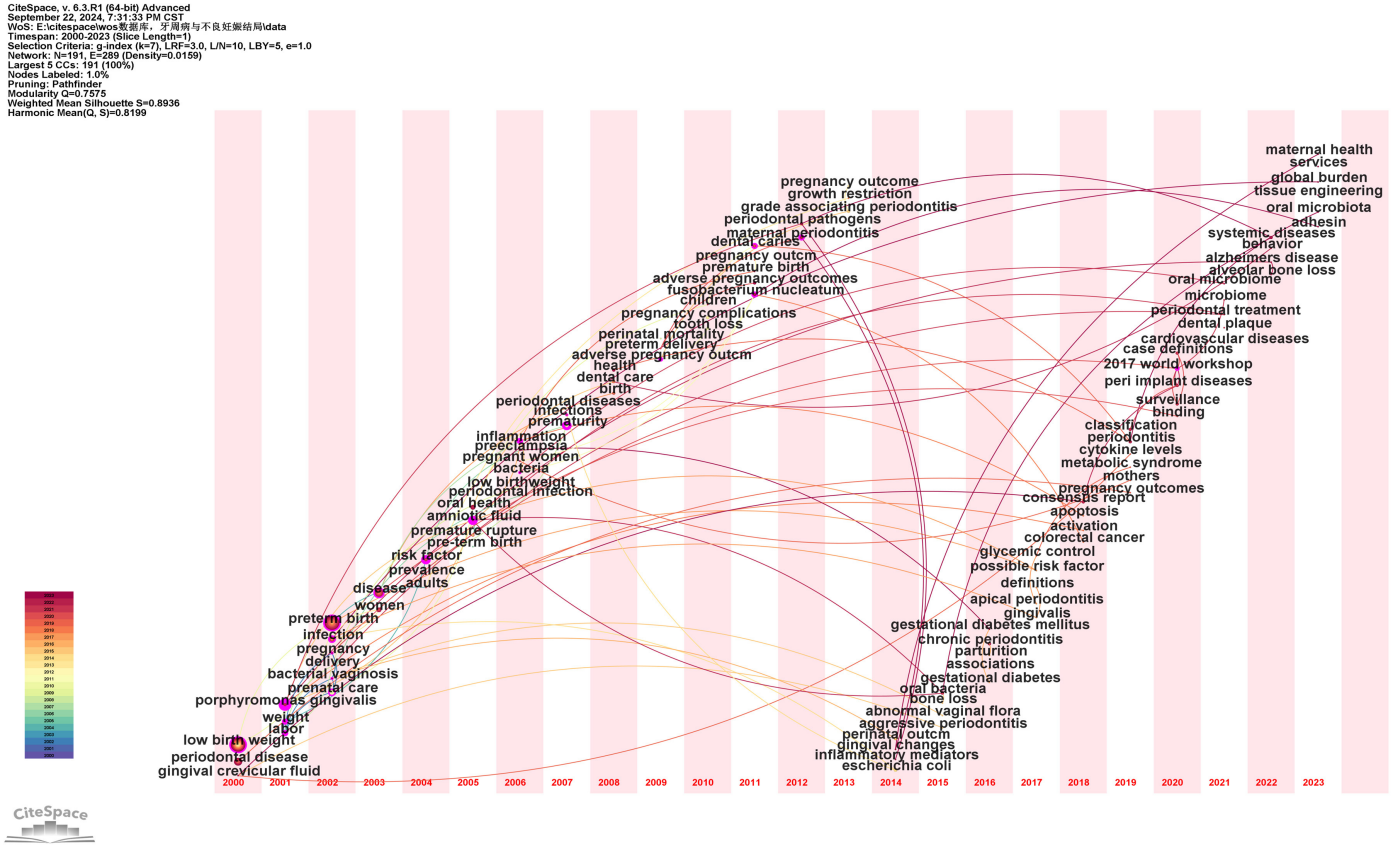
Keyword time zone map. In this figure, nodes represent keywords, with the size of each node indicating the frequency of the keyword, and the color representing the time of the keyword's first appearance. Links between nodes indicate co-occurrence relationships between the keywords.

Using the above data, a keyword time zone analysis was conducted, illustrating the distribution and evolving trends of keywords across temporal and spatial dimensions. As shown in Figure 12, throughout the past 20 years, research on the relationship between PD and APOs has deepened and expanded continuously. The research focus has evolved from early foundational pathology and clinical observational studies to more complex areas such as oral microbiome, vaginal microbiome, inflammatory factors, gene expression, and preventive and therapeutic aspects of oral health. These studies provide valuable insights into how PD affects APOs and highlight future research directions (see Supplementary Table 11).

#### 3.4.5 Hotspots and cutting-edge topics

**Figure 13:**
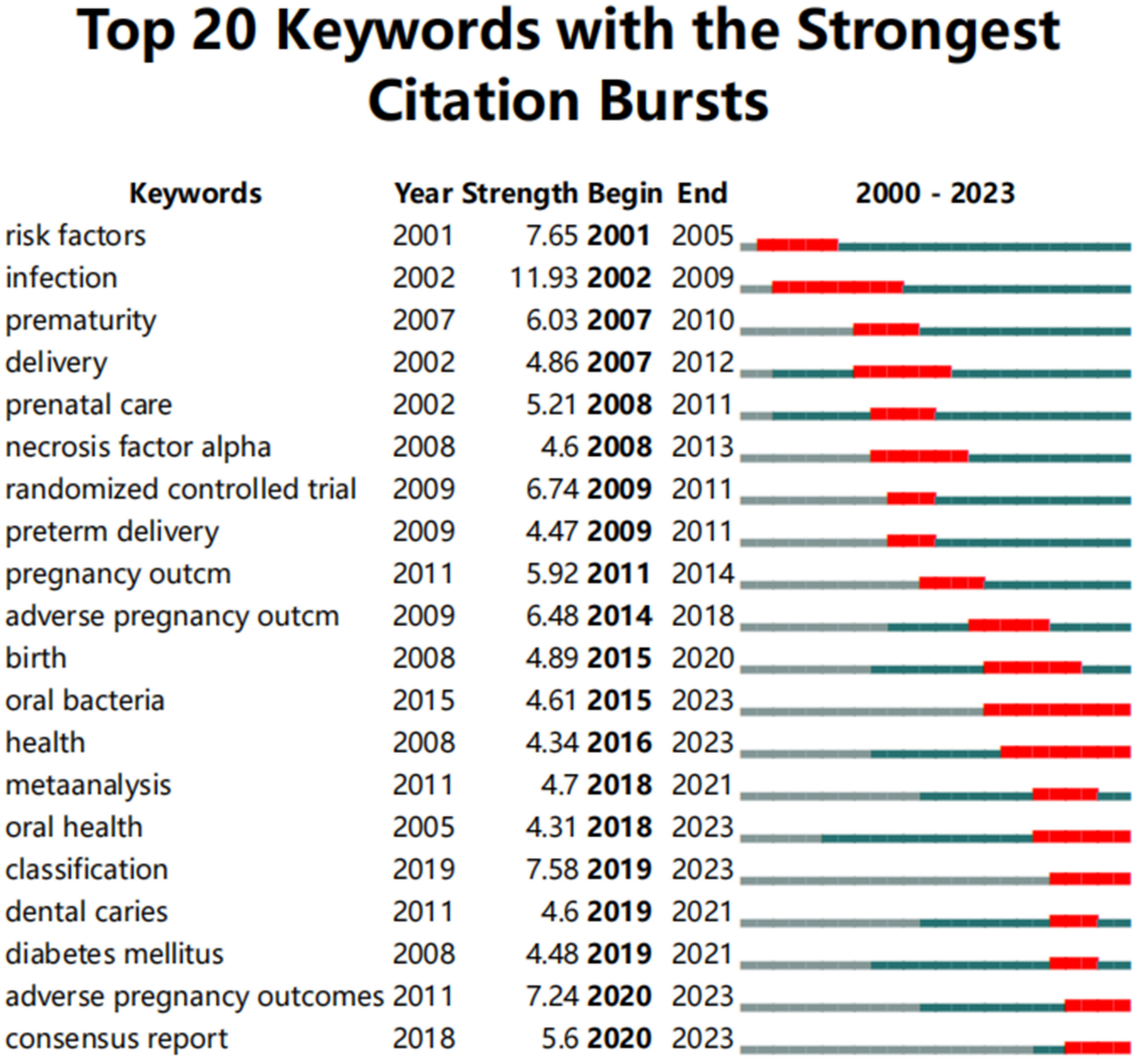
Top 20 keywords with the strongest citation bursts. The red line represents years when keywords burst, and the blue line indicates years when keywords were used less frequently. Burst strength reflects the occurrence times of keywords in a certain period. Each keyword burst lasts for a minimum duration of 3 years.

The emergence of sudden keywords is related to the repeated appearance of specific terms by scholars in a defined timeframe (42). As shown in Figure 13, the analysis conducted using CiteSpace reveals the top 20 keywords with the strongest citation bursts. Overall, the burst durations of these 20 keywords spanning from 1 to 8 years, with burst intensity varies between 4.31 and 11.93. The keyword with the strongest burst (intensity = 11.93) is “infection,” with the burst citation occurring between 2002 and 2009. The second strongest burst (intensity = 7.65) is “risk factors,” occurring from 2001 to 2005. Both keywords have garnered significant attention since their initial appearance, quickly becoming frequently cited terms. This reflects the academic community's heightened focus on PD as a major source of infection and risk during pregnancy, further prompting in-depth discussions on the inflammatory mechanisms and risk management concerning pregnancy outcomes. Additionally, the keyword “oral bacteria” has the longest burst duration. The earliest emergent keyword in this study is “risk factors,” while “adverse pregnancy outcomes” and “consensus report” are the latest additions since 2020. A comprehensive analysis of these along with currently frequently cited keywords such as “oral bacteria,” “health,” “oral health,” and “classification” indicates that they may represent the current hot topics and future trends in the research on PD and APOs.

### 3.5 Systematic search analysis

**Figure 14:**
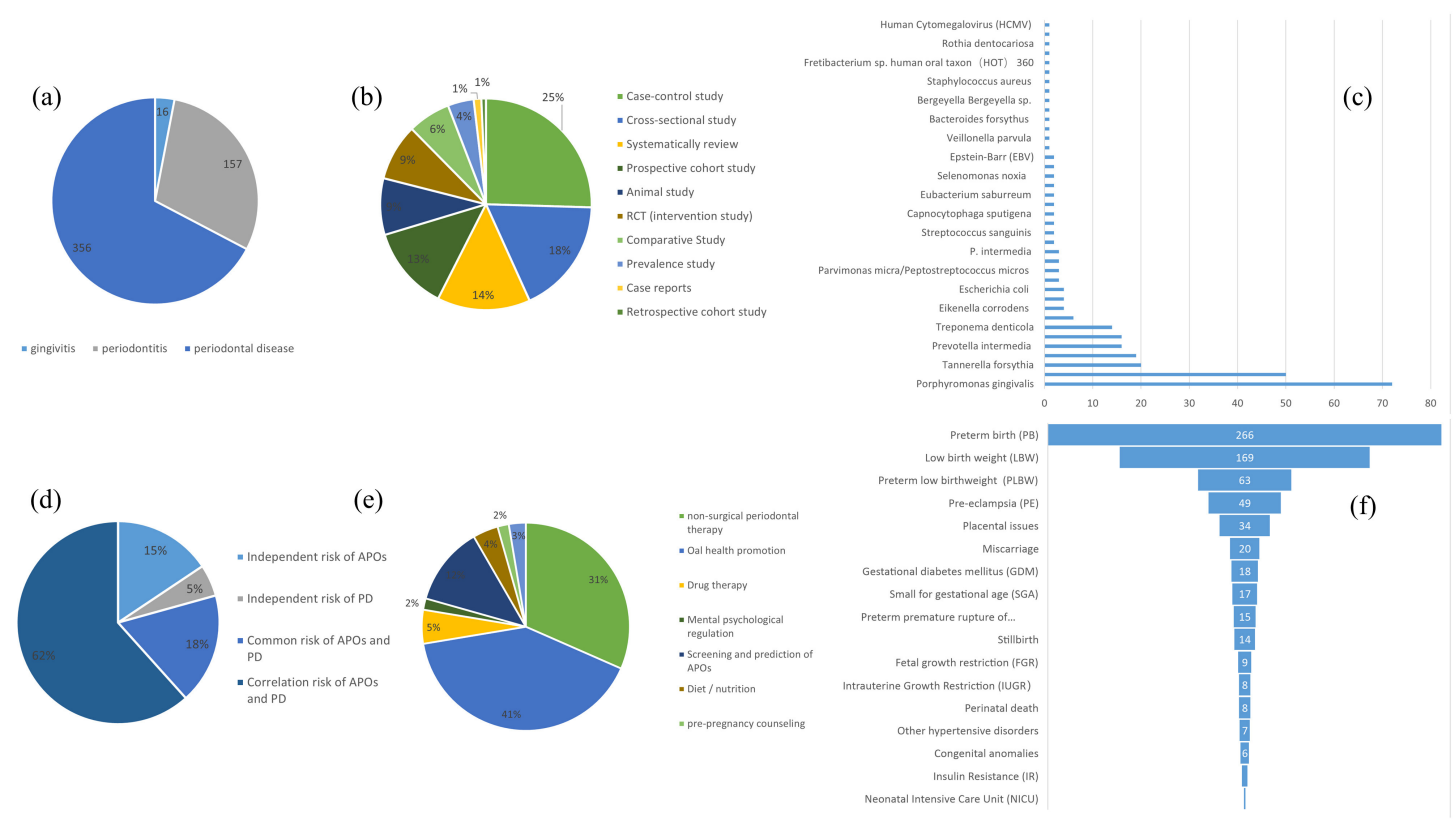
**(A)** The number of adverse pregnancy outcomes (APOs) associated with gingivitis or periodontitis and periodontal disease (including both gingivitis and periodontitis, without significant distinction), respectively. **(B)** Quantitative statistics for each study design on PD and APOs. **(C)** Articles on the role of each key pathogen in APOs Types of studies and quantitative statistics. **(D)** The number of studies on risk (including independent risk of APOs, independent risk of PD, common risk of APOs and PD, and correlation risk of APOs and PD). **(E)** Quantitative statistics on ways to prevent APOs. **(F)** Classification of articles on the correlation between each type of APO and periodontal disease.

As a complement to the bibliometric analysis, we conducted a systematic screening of the 932 articles identified in the WoSCC database. The screening process was based on their titles, abstracts, keywords, and full texts, followed by six points of systematic analysis: (a) Statistical analysis of studies on gingivitis/periodontitis/PD and APOs: As shown in Figure 14A, 16 studies were identified as related to gingivitis and APOs, 157 studies to periodontitis and APOs, and 356 studies to PD, including both gingivitis and periodontitis, related to APOs. A detailed list of these articles is provided in Sheet 1 in Supplementary Table 14. (b) Analysis of articles by study design: According to Figure 14B, research designs investigating the relationship between PD and APOs include Case-control studies (109), Cross-sectional studies (76), Systematic reviews (61), Prospective cohort studies (55), Animal studies (37), RCT (intervention study) (37), Comparative studies (28), Prevalence studies (17), Case reports (5), and Retrospective cohort studies (3). Case-control studies were the most common. Additionally, the top three most-cited articles in the first six study designs were tabulated, as shown in Table 1. Detailed lists and content analyses (e.g., animal models/population characteristics, outcome indicators, main conclusions) for these study types are included in Supplementary Table 15. (c) Statistical analysis of key oral bacteria in APOs: As shown in Figure 14C, oral bacteria investigated in more than 10 studies include *Porphyromonas gingivalis* (72 studies), *Fusobacterium nucleatum* (50 studies), Tannerella forsythia, Aggregatibacter actinomycetemcomitans, Prevotella intermedia, Campylobacter rectus, and Treponema denticola. Most studies focused on chronic periodontitis-associated bacteria, particularly those from the red and orange microbial complexes in subgingival plaque. Detailed lists of these bacteria-related studies are provided in Sheet 2 in Supplementary Table 14. (d) Statistical analysis of risk factor studies: As shown in Figure 14D, risk factors were categorized into four groups: independent risk factors for APOs (114), independent risk factors for PD (37), shared risk factors for PD and APOs (129), and factors associated with the PD-APOs relationship (450). Research on risk factors aids in early identification, prevention, and treatment efforts. Further details are provided in Sheet 3 in Supplementary Table 14. (e) Statistical analysis of studies on the role of non-surgical periodontal therapy in APO prevention: According to Figure 14E, interventions were roughly classified into non-surgical periodontal therapy, oral health promotion, drug therapy, mental and psychological regulation, screening and prediction of APOs, and diet/nutrition. Research specifically on the prevention of APOs through non-surgical periodontal therapy included 72 studies. Detailed information is available in Sheet 4 in Supplementary Table 14. (f) Statistical analysis of APO types and their relationship with PD: As shown in Figure 14F, studies on PD and APOs primarily focus on delivery-related issues and fetal growth and development issues, such as preterm birth (PB), low birth weight (LBW), and preterm low birth weight (PLBW). Research on pregnancy complications, such as pre-eclampsia (PE) and gestational diabetes mellitus (GDM), is increasing. However, fewer studies address fetal and neonatal deaths (e.g., stillbirth, perinatal death) and neonatal issues [e.g., neonatal intensive care unit (NICU) admissions, insulin resistance (IR)]. The detailed lists of studies related to these APO types are provided in Sheet 5 in Supplementary Table 14.

**Table 1:** For specific research design [animal study, cross-sectional study, prospective cohort study, case-control study, RCT (intervention study), systematic review], the first three articles with the highest number of citations were collated.

**Study type**	**Title**	**DOI**	**First author**	**Year**	**Journal**	**Citations**
Animal study	*Fusobacterium nucleatum* induces premature and term stillbirths in pregnant mice: implication of oral bacteria in preterm birth	doi: 10.1128/IAI.72.4.2272-2279.2004	Han, YPW	2004	Infection and Immunity	311
	Transmission of diverse oral bacteria to murine placenta: evidence for the oral microbiome as a potential source of intrauterine infection	doi: 10.1128/IAI.01395-09	Fardini, Yann	2010	Infection and Immunity	191
	Maternal LPS induces cytokines in the amniotic fluid and corticotropin releasing hormone in the fetal rat brain	doi: 10.1152/ajpregu.00664.2003	Gayle, DA	2004	American Journal of Physiology-Regulatory Integrative and Comparative Physiology	177
Cross-sectional study	Factors related to utilization of dental services during pregnancy	doi: 10.1111/j.1600-051X.2005.00739.x	Al Habashneh, R	2005	Journal of Clinical Periodontology	93
	Pregnancy and oral health: utilization of dental services during pregnancy in northern Greece	doi: 10.1080/00016340701371413	Dinas, Konstantinos	2007	Acta Obstetricia et Gynecologica Scandinavica	60
	Is there an association between periodontal disease, prematurity and low birth weight? A population-based study	doi: 10.1111/j.1600-051X.2005.00759.x	Lunardelli, AN	2005	Journal of Clinical Periodontology	59
Prospective cohort study	Periodontal infection and preterm birth - results of a prospective study	doi: 10.14219/jada.archive.2001.0299	Jeffcoat, MK	2001	Journal of the American Dental Association	338
	Progressive periodontal disease and risk of very preterm delivery	doi: 10.1097/01.AOG.0000190212.87012.96	Offenbacher, S	2006	Obstetrics and Gynecology	240
	A prospective study to investigate the relationship between periodontal disease and adverse pregnancy outcome	doi: 10.1038/sj.bdj.4811620	Moore, S	2004	British Dental Journal	157
Case-control study	Maternal periodontal disease and preterm low birthweight: case-control study	doi: 10.1177/154405910208100505	Davenport, ES	2002	Journal of Dental Research	191
	Periodontal disease and upper genital tract inflammation in early spontaneous preterm birth	doi: 10.1097/01.AOG.0000139836.47777.6d	Goepfert, AR	2004	Obstetrics and Gynecology	140
	Periodontitis is associated with preeclampsia in pregnant women	doi: 10.1902/jop.2006.050020	Contreras, A.	2006	Journal of Periodontology	119
RCT (intervention study)	Treatment of periodontal disease and the risk of preterm birth	doi: 10.1056/NEJMoa062249	Michalowicz, Bryan S.	2006	New England Journal of Medicine	360
	Periodontal therapy may reduce the risk of preterm low birth weight in women with periodontal disease: a randomized controlled trial	doi: 10.1902/jop.2002.73.8.911	López, NJ	2002	Journal of Periodontology	350
	Periodontal disease and preterm birth: results of a pilot intervention study	doi: 10.1902/jop.2003.74.8.1214	Jeffcoat, MK	2003	Journal of Periodontology	256
Systematical review	The effects of waterpipe tobacco smoking on health outcomes: a systematic review	doi: 10.1093/ije/dyq002	Akl, Elie A.	2010	International Journal of Epidemiology	442
	Periodontal disease and adverse pregnancy outcomes: a systematic review	doi: 10.1111/j.1471-0528.2005.00827.x	Xiong, X	2006	BJOG-An International Journal of Obstetrics and Gynecology	369
	Health effects associated with waterpipe smoking	doi: 10.1136/tobaccocontrol-2014-051908	El-Zaatari, Ziad M.	2015	Tobacco Control	261

## 4 Discussion

### 4.1 General information

This study includes a total of 932 English articles published over the past 23 years in the WOSCC database, related to PD and APOs. A comprehensive analysis was conducted from various aspects, including the number of publications, countries, institutions, authors, journals, citation counts, keywords, and trending topics. Since 2000, the research field has undergone several growth phases, with particularly rapid expansion after 2019, peaking at 75 articles in 2023. This indicates that research in this area is receiving increasing attention and support and will likely remain a key topic in future years.

Scientific collaboration refers to researchers working together to create new scientific knowledge. This collaboration can occur at various levels, including individual, institutional, and international cooperation. In evaluating the importance of nodes within collaborative networks, both social connections and academic output are crucial, with betweenness centrality (BC) serving as a key measure of social connectivity. A BC value exceeding 0.10 illustrates that the node is a central one, playing a comparatively significant and influential role in the research. (I) At the national collaboration level, as shown in Figure 3 and Supplementary Table 3, the top three countries by number of publications are the USA (*n* = 355, 38.09%), Brazil (*n* = 85, 9.12%), and India (*n* = 59, 6.33%). The top three countries by betweenness centrality (BC) are the USA (0.77), Australia (0.14), and Canada (0.13). The USA contributes more than one-third of the total publications and holds the highest BC value, indicating its significant influence in this field. In contrast, Brazil and India have lower BC values of 0.02 and 0.01, respectively, suggesting limited collaboration with international peers, potentially due to economic constraints that hinder their impact. (II) At the institutional collaboration level (Figure 4; Supplementary Table 4), the top three institutions by publication volume are the University of North Carolina (*n* = 34), the University of North Carolina Chapel Hill (*n* = 33), and Columbia University (*n* = 28). Institutions with a betweenness centrality (BC) value >0.1 include Columbia University (0.16) and the National Institutes of Health (NIH)—USA (0.11). Columbia University not only has a relatively high publication output (*n* = 28) but also holds a significant BC value (0.16), indicating its prominent role in both academic output and as a key connector within the collaboration network. Additionally, the NIH, a government institution focused on health and biomedical research, has a BC value of 0.11, demonstrating its influence and central position in the field, even though its publication volume does not rank in the top three. Although the University of North Carolina and its Chapel Hill branch rank high in publication volume, their BC values do not exceed 0.1, suggesting a relatively weaker bridging role within the collaboration network or more concentrated collaborations within smaller circles. (III) At the author collaboration level, only five authors have published more than five papers: Steven Offenbacher (*n* = 24), Boggess, KA (*n* = 11), Beck, JD (*n* = 9), Papapanou, Panos N (*n* = 6), and Ahn, Ki Hoon (*n* = 5; see Supplementary Table 5). The highest betweenness centrality (BC) value is 0.01 for Steven Offenbacher, indicating that most researchers tend to work independently with limited collaborative publications, which may contribute to the low network density. This could be attributed to factors such as geographic distribution, disciplinary boundaries, or resource allocation. Nevertheless, a significant research network exists between key authors, particularly Steven Offenbacher, Boggess, KA, and Beck, JD (Figure 5). Steven Offenbacher, in particular, pioneered research in this field by first reporting in 1996 that women with PD had a nearly seven times increased risk of PB and LBW compared to controls (16). This study marked the emergence of a new research area on PD and APOs, in which Offenbacher has remained active through 2023. Additionally, eight authors have a co-citation centrality exceeding 0.10 (see Supplementary Table 5), playing a bridging role in the research network. Steven Offenbacher remains the most cited author, further underscoring his significant contribution to the field and his leadership in PD and APOs research.

The co-citation analysis of journals indicates that *Journal of Periodontology* is the most frequently cited journal in the field of PD, establishing its central role in this domain. Other highly cited journals, such as *Journal of Clinical Periodontology* and *Journal of Dental Research*, further demonstrate their significant influence in PD research. Additionally, *American Journal of Obstetrics and Gynecology* and *Obstetrics and Gynecology* are the most frequently cited journals in the field of obstetrics and gynecology, underscoring their leadership in maternal health research. Journals such as *BJOG: An International Journal of Obstetrics & Gynecology* also show notable citation frequency, reflecting their influence in this area. The journal with the highest betweenness centrality (BC) is *Acta Odontologica Scandinavica* (0.55), indicating its pivotal role in bridging different research fields or journals and enhancing its centrality within the academic network. This analysis not only highlights the key academic journals in PD and obstetrics but also underscores the interdisciplinary nature of PD and APOs, showing the high scientific value and broad impact of this Research Topic.

### 4.2 Knowledge base

Co-citation analysis evaluates the strength of connection between papers, and the knowledge base comprises frequently cited references. This study includes the five most co-cited papers in the “PD and APOs” field (see Supplementary Table 7). The most cited paper is Michalowicz et al.'s 2006 study, “*Treatment of periodontal disease and the risk of preterm birth,”* published in *The New England Journal of Medicine*. The keywords of this study include “periodontal disease,” “preterm birth,” “low birth weight,” and “fetal growth restriction.” The study uses a randomized controlled trial (RCT) to assess the effects of non-surgical PD treatment. A total of 823 pregnant women (13–17 weeks gestation) were randomly divided into two groups: 413 received treatment before 21 weeks of pregnancy, and 410 received treatment postpartum. The treatment group underwent scaling and root planing, followed by monthly tooth polishing and oral hygiene instructions, while the control group received only monthly oral examinations and guidance. The primary outcome was gestational age (GA) at delivery, while secondary outcomes included birth weight and the proportion of small-for-gestational-age (SGA) infants. The results showed no statistically significant differences between the treatment and control groups in preterm birth rates, birth weight, SGA proportions, spontaneous abortions, or stillbirths. Furthermore, the treatment group showed significant improvements in PD markers, such as probing depth (PD) and clinical attachment loss (CAL). This study concludes that while treating maternal PD improves periodontal health and is safe, it does not significantly affect rates of PB, LBW, or FGR (27).

The second study is by Jeffcoat et al., titled “*Periodontal disease and preterm birth: results of a pilot intervention study,”* published in 2003 in the *Journal of Periodontology*. The keywords for this publication include “Periodontal Disease,” “Spontaneous preterm birth,” “Intervention Study,” and “Metronidazole.” This study evaluates the effectiveness of different periodontal treatment methods in reducing preterm birth rates through a randomized controlled trial (RCT). A total of 366 pregnant women with periodontitis, at 21 to 25 weeks of gestation, were stratified based on two factors: (1) a history of preterm birth (< 35 weeks), and (2) a body mass index (BMI) < 19.8 or the presence of bacterial vaginosis assessed via Gram stain. Participants were randomly assigned to one of three groups: (1) dental prophylaxis with a placebo capsule, (2) scaling and root planing (SRP) with a placebo capsule, and (3) SRP with metronidazole capsules (250 mg three times a day for 1 week). All participants received oral hygiene instructions and home care supplies (toothbrush, floss, and fluoride toothpaste). The prophylaxis group underwent supragingival scaling and rubber cup polishing, while the SRP groups received standard clinical scaling and root planing. The primary outcome was the rate of preterm birth. The results indicated a significant reduction in preterm birth rates in the SRP plus placebo group compared to the prophylaxis group. However, the preterm birth rate was higher in the SRP plus metronidazole group (*P* = 0.02). This trial suggests that SRP may reduce the preterm birth rate among pregnant women with periodontitis. The addition of metronidazole did not improve pregnancy outcomes (25).

The third study is by X. Xiong et al., published in 2006 in *BJOG: An International Journal of Obstetrics and Gynecology*, titled “*Periodontal disease and adverse pregnancy outcomes: a systematic review.”* The keywords for this publication include “Periodontal disease,” “adverse pregnancy outcomes,” “systematic review,” and “association.” This study aims to examine the existing evidence regarding the relationship between PD and APOs, identifying 25 studies (13 case-control studies, 9 cohort studies, and 3 controlled trials). These studies focus on outcomes such as preterm birth, low birth weight, fetal growth restriction, miscarriage, and preeclampsia. Among the selected studies, 18 indicated an association between PD and an increased risk of APOs (odds ratios ranging from 1.10 to 20.0), while seven found no evidence of an association (odds ratios ranging from 0.78 to 2.54). Three clinical trials demonstrated that oral prophylaxis and periodontal treatment could reduce the incidence of preterm low birth weight by 57% [pooled relative risk (RR) 0.43; 95% confidence interval (CI) 0.24–0.78] and preterm birth by 50% (RR 0.5; 95% CI 0.20–1.30). This study indicates that while most research supports the notion that PD is a risk factor for APOs, the heterogeneity in definitions of PD and APOs across different studies, as well as potential confounding factors, prevent the formulation of definitive conclusions (19).

The fourth study is by Offenbacher et al., titled “*Effects of periodontal therapy on rate of preterm delivery: a randomized controlled trial*” published in 2009 in *Obstetrics and Gynecology*. The keywords associated with this publication include “periodontal disease,” “preterm delivery,” “randomized controlled trial,” and “periodontal therapy.” This study recruited a total of 1,806 pregnant women with PD. Through a randomized, treatment-blind, controlled clinical trial, participants were randomly assigned to either the periodontal therapy group (receiving treatment during mid-pregnancy and early pregnancy) or the delayed treatment group (receiving treatment after delivery). The treatment group underwent periodontal therapy during mid-pregnancy and early pregnancy, which included scaling and root planing (SRP) above and below the gumline, full mouth polishing, and oral hygiene instruction. The delayed treatment group received the same periodontal therapy after delivery. The primary outcome measured was the rate of preterm delivery at < 37 weeks of gestation, while secondary outcomes included rates of preterm birth at < 35 weeks, average newborn birth weight, and rates of neonatal morbidity and mortality. The results indicated no significant differences between the treatment group and the control group regarding rates of preterm delivery at < 37 weeks, rates of preterm delivery at < 35 weeks, average birth weight, and rates of neonatal morbidity and mortality. Additionally, the periodontal condition of the delayed treatment group significantly worsened, while the treatment group showed some improvement in periodontal condition, although a substantial proportion of participants did not achieve periodontal health standards. This study suggests that singular periodontal treatment may be insufficient to effectively manage periodontal inflammation during pregnancy and prevent preterm birth (28).

The fifth study is by López et al., titled “*Periodontal therapy may reduce the risk of preterm low birth weight in women with periodontal disease: a randomized controlled trial*” published in 2002 in the J*ournal of Periodontology*. The keywords associated with this publication include “periodontal disease,” “preterm low birth weight,” “randomized controlled trial,” and “periodontal therapy.” The study involved pregnant women aged 18–35 years with PD, totaling 400 participants who were randomly divided into two groups: the experimental group (200 participants) received periodontal treatment before the 28th week of pregnancy, while the control group (200 participants) received treatment after delivery. The experimental group underwent periodontal treatment before the 28th week of pregnancy, which included plaque control instruction, scaling, and root planing. The control group received the same treatment after delivery. Historical and current pregnancy data, along with known risk factors, were obtained from patient records and interviews. The primary outcome assessed was delivery before 37 weeks of gestation or an infant birth weight of < 2,500 grams. The results indicated that periodontal disease was the strongest associated factor for preterm low birth weight (PLBW; OR 4.70, 95% CI 1.29–17.13). Other significant factors included a history of PLBW (OR 3.98, 95% CI 1.11–14.21), fewer than six prenatal visits (OR 3.70, 95% CI 1.46–9.38), and inadequate maternal weight gain (OR 3.42, 95% CI 1.16–10.03). Additionally, women with PD had a significantly higher incidence of PLBW compared to those without PD, with the risk of PLBW increasing with the severity of PD. The study concluded that PD appears to be an independent risk factor for PLBW, and that periodontal therapy can significantly reduce the incidence of PLBW in women with PD, emphasizing the importance of periodontal health during pregnancy and recommending early periodontal treatment to improve pregnancy outcomes (26).

A brief bibliometric analysis of the five studies mentioned yields the following results: (I) The total citation counts for these articles are 96, 64, 63, 61, and 57, respectively (see Supplementary Table 7). The most highly cited article is Michalowicz BS, 2006, published in *The New England Journal of Medicine*, indicating its broad recognition and influence within the field. (II) The betweenness centrality (BC) values of these studies are 0.77, 0.1, 0.04, 1, and 0.86, respectively (see Supplementary Table 7), with the highest BC value belonging to Offenbacher S, 2009, in *Obstetrics and Gynecology*, suggesting its pivotal role in bridging various topics within the literature network. (III) The authors of these five studies—Michalowicz, Jeffcoat, X Xiong, Offenbacher, and López—rank among the top six most co-cited authors. (IV) The journals in which these articles are published include *The New England Journal of Medicine, Journal of Periodontology, Bjog-an International Journal of Obstetrics and Gynecology, Obstetrics and Gynecology*, and *Journal of Periodontology*. Analysis of Figure 8 and Supplementary Table 8 reveals that two of these papers [by Jeffcoat et al. (25) and López et al. (26)] were published in the *Journal of Periodontology*, one of the leading journals in the field of periodontology, consistent with our previous findings. (V) A summary of the frequently occurring keywords in these studies shows repeated terms such as “periodontal disease,” “preterm birth,” and “low birth weight.” Given the publication timeline of these studies, spanning from 2002 to 2009, and in combination with the keyword timeline visualization and keyword time-zone analysis in this study (Figures 11, 12), it is evident that from 2000 to 2010, research in the PD and APOs field transitioned from basic research to more advanced studies. The keywords gradually shifted from “low birth weight” and “periodontal disease” to “preterm birth” and “pregnancy complications.” This reflects the evolution of research focus from the basic etiology of PD and its effects on LBW to a broader investigation of PD's impact on various pregnancy complications, further supporting the reliability and significance of this study.

A comparison of the five studies in terms of research type, study size and scope, research design and interventions, and conclusions reveals both consistency and differences: (I) In terms of research type, most of the studies are RCTs aimed at evaluating the effect of periodontal therapy on pregnancy outcomes. (II) Regarding study size and scope, the trials by Michalowicz et al. (27) and Offenbacher et al. (28) involved larger sample sizes with 823 and 1,806 pregnant women, respectively. In contrast, the studies by Jeffcoat et al. (25) and López et al. (26) were pilot or smaller-scale trials with 366 and 400 participants, respectively. Xiong et al. (19) conducted a systematic review that summarized findings from multiple studies. (III) In terms of research design and interventions, the studies by Michalowicz et al. (27) and Offenbacher et al. (28) were designed as RCTs that primarily assessed the impact of non-surgical periodontal therapy on PB. Similarly, the studies by Jeffcoat et al. (25) and López et al. (26) also adopted RCT designs, but Jeffcoat et al. (26) explored the additional effect of adjunctive metronidazole therapy. While their results showed that adjunctive metronidazole did not improve pregnancy outcomes, this highlights the need for future studies to explore various combinations of interventions to identify the most effective treatment approach for PD during pregnancy. Additionally, the impact of medications on both the fetus and mother during pregnancy should be considered. Although drugs such as metronidazole have been proposed for preventing late pregnancy complications, their efficacy remains controversial (7). Therefore, when using systemic antibiotics during pregnancy, careful consideration should be given to the timing and choice of medication. (IV) In terms of research outcomes, most studies [e.g., Michalowicz et al. (27), Offenbacher et al. (28)] found no significant effect of periodontal therapy on PB and LBW. However, some studies [e.g., López et al. (26)] demonstrated significant positive outcomes. Recent analysis by Bobetsis et al. (20), which included 15 RCTs conducted globally to evaluate the impact of mid-pregnancy non-surgical periodontal interventions on APOs, showed that most periodontal interventions improved periodontal parameters but had no significant effect on APOs. One plausible explanation for these discrepancies is the lack of collaboration between institutions across different countries, leading to inconsistencies in the definitions of PD and APOs as well as the influence of confounding factors (19). This suggests the need for an international consortium to ensure uniform methods and study designs, avoid unnecessary duplication, and provide the most effective and optimal results in this field. Another explanation could be related to the mechanism by which periodontal pathogens cause APOs. Periodontal therapy during pregnancy may not eliminate oral pathogens that had already reached the placenta-fetus interface early in pregnancy, or the treatment may not mitigate exposure to the intrauterine infection pathways triggered by “key pathogens” (7, 20). Thus, the improvement in periodontal health indicators may not have effectively alleviated the symptoms of APOs. More research and innovative practices are needed to determine how to effectively intervene in the risk of APOs caused by PD. Moreover, it should be noted that lower citation counts for certain papers do not necessarily reflect poor research quality. It may also be due to the lower impact of the journal or the more recent publication date.

### 4.3 Research hotspots between PD and APOs

Keywords are essential for retrieving articles for analysis comprehensively. They encapsulate the main ideas and core essence of an article, effectively highlighting the research hotspots in a particular field (58). This study also conducted keyword clustering analysis to summarize and organize the specific research areas reflected by the keywords. By overlaying a visualization of the temporal evolution of keywords in the PD and APOs field, the study provides insights into the shifting research focus over time, which can better inform future research directions.

The top four most frequent keywords are “preterm birth,” “low birth weight,” “periodontal disease,” and “risk” (see Supplementary Table 9), which is consistent with the examination of the five most frequently cited articles within the knowledge base. Although our search strategy included terms such as “miscarriage,” “stillbirth” and “postpartum complications,” these keywords are relatively rare in the study results. This may indicate limited research on PD in these subfields, with insufficient supporting evidence. However, given that issues such as miscarriage, stillbirth, and postpartum complications have severe impacts on women's physical and mental health (59–61), future studies may yield more valuable data and conclusions to better support women's health. Additionally, with BC = 0.2 set as the threshold, the keyword “risk factor^*^” appeared in both 2001 and 2004 (see Supplementary Table 9; Figure 9). Considering that “risk” is a high-frequency keyword and “risk factors” is the earliest burst keyword in the subsequent keyword burst analysis (Figure 13), it highlights that risk factors are not only a central theme in PD and APOs research but also have a vital part in linking different topics within the literature network. This likely encompasses multiple research subfields, including periodontitis, PB, and LBW. PD refers to the chronic inflammation and degeneration of periodontal tissues caused by bacterial infections (2). Theoretically, any factor that promotes plaque accumulation can contribute to the onset of PD, including inadequate dental care, malocclusion, and alignment issues. Additionally, individual risk factors encompass gender, stress, smoking, alcohol intake (lifestyle), obesity, diabetes, metabolic syndrome, dietary calcium and vitamin D deficiency, osteoporosis and genetic components (62). The risk factors for APOs cover various maternal, fetal, and environmental aspects. Modifiable factors include smoking, alcohol consumption, and obesity; medically treatable factors include PD, vaginitis, and anemia; closely monitored factors include hypertension and diabetes; and those requiring prenatal diagnosis include advanced maternal age, adverse pregnancy history, and family history of genetic disorders (63–67). As briefly discussed in the introduction, the bidirectional and complex relationship between PD and APOs is not a simple cause-effect interaction. The analysis of risk factors reveals shared modifiable factors. Grant et al. propose that, given PD often precedes other negative health consequences, the diagnosis of PD can serve as a warning to patients, indicating that lifestyle changes may minimize the likelihood of negative health outcomes (68). This perspective opens new avenues for preventing APOs and other systemic diseases.

Based on the keyword clustering results (Figure 10; Supplementary Table 10), the largest cluster is “*Porphyromonas gingivalis* (#0),” which includes keywords such as “*Porphyromonas gingivalis*,” “coronary heart disease,” “C-reactive protein,” “bone mineral density,” and “atherosclerosis.” This cluster centers on *Porphyromonas gingivalis* and covers several keywords related to systemic diseases, reflecting the potential association between PD and chronic systemic conditions, particularly coronary heart disease, atherosclerosis, and osteoporosis. Extensive research has already explored the link between *Porphyromonas gingivalis* and cardiovascular disease (69, 70). Studies have also shown that proper periodontal care can decrease the occurrence of cardiovascular mortality, heart failure, and myocardial infarction (71, 72). Given the association between PD and APOs, special attention should be given to pregnant women with PD who also have other systemic diseases during pregnancy. More proactive treatment and care strategies should be implemented to reduce the incidence of APOs.

The keyword time-zone analysis and timeline view are effective in depicting existing research focus and emerging trends over a specified period. As shown in Figures 11, 12, these developments are described chronologically: (I) 2000 to 2005: Foundational Research Stage. During this period, keywords such as “low birth weight,” “periodontal disease,” “cardiovascular disease,” and “gingival crevicular fluid” dominated. Research primarily focused on the basic etiology of PD and its impact on LBW. Simultaneously, the relationship between PD and cardiovascular disease gained attention as an early research direction. The studies during this time mainly involved fundamental pathology and clinical observations, laying the groundwork for understanding the link between PD and APOs like PB and LBW. (II) 2006 to 2010: In-Depth Investigation of PD and Pregnancy Outcomes. This period saw increased focus on keywords related to APOs, such as “preterm birth” and “pregnancy complications.” The keyword “inflammation” also emerged as a significant research area, highlighting how PD might affect pregnancy outcomes through systemic inflammatory responses. Research expanded beyond the direct effects of PD to examine how periodontal inflammation and immune responses influence outcomes like PB and FGR. PD began to be acknowledged as a potential risk factor during pregnancy, particularly through inflammation-mediated mechanisms. (III) 2011 to 2015: Interdisciplinary Research on Microbiology and Systemic Effects. This phase introduced keywords like “*Fusobacterium nucleatum*” and “oral bacteria,” reflecting the growing interest in the impact of oral microbiota on pregnancy health. The appearance of terms like “bacterial vaginosis” indicated a broader scope of research, incorporating the influence of various microbial infections on pregnancy outcomes. (IV) 2016 to 2020: Emerging Hotspots and Cross-Disciplinary Integration. Keywords such as “oral microbiome,” “cytokine levels,” and “metabolic syndrome” emerged during this period. Studies increasingly focused on the complex interactions between PD and pregnancy complications, including interdisciplinary directions like microbiome research and metabolic syndrome. The research emphasis shifted to how PD, through its effects on the oral microbiome and systemic metabolic processes, could exacerbate pregnancy complications. Evidence grew supporting PD's role in APOs through immune and metabolic pathways (7, 20, 21). (V) 2021 to 2023: Cutting-Edge Research on Microbiomes and Maternal-Fetal Health. Recent keywords such as “oral microbiota,” “global burden,” and “tissue engineering” suggest a shift toward more forward-looking and globally relevant research questions. “Oral microbiome” has become a key focus in recent years, reflecting an increasing interest in the cross-relationship between oral and systemic health. Current studies now encompass a global health perspective, investigating the burden of PD across different populations and its broader implications beyond traditional associations with pregnancy outcomes.

Based on the keyword burst analysis (Figure 13), “oral bacteria” has the longest burst duration, followed by “health.” More recently, keywords like “adverse pregnancy outcomes” and “consensus report” have emerged as new bursts since 2020. Additionally, keywords that continue to be frequently cited include “oral bacteria,” “health,” “oral health,” “classification,” “adverse pregnancy outcomes,” and “consensus report.” In conjunction with the keyword time-zone analysis shown in Figure 11, it can be predicted that “oral bacteria,” “health,” “oral health,” “adverse pregnancy outcomes,” and “consensus report” will continue to experience sustained bursts in the future. The pathogenic mechanism model of PD and APOs proposed by Bobetsis et al. (20) is expected to continue serving as an authoritative guide for both research and clinical practice, especially regarding the prevention and treatment of PD, and the relationship between the oral microbiome and systemic health. This model may also facilitate increased international collaboration, contributing to greater research standardization and the production of higher-quality studies.

Emerging keywords such as “oral microbiota” no longer refer solely to the bacterial populations in the oral cavity but also encompass the effects of various microbial metabolites on the host. Current evidence indicates that the oral cavity hosts ~770 microbial species, with the composition and diversity of the oral microbiome being influenced by various environmental factors, such as pH levels, anaerobic conditions, nutrient availability, and hormone fluctuations. Under normal physiological conditions, a balanced homeostasis exists among these microorganisms. However, dysbiosis in the oral microbiome is a major cause of oral diseases, such as caries and chronic periodontitis (73). Moreover, oral microbial imbalance is linked to the pathogenesis of systemic conditions, including diabetes, cardiovascular disease, and APOs (74). Recent evidence suggests that the composition of the oral microbiome undergoes pathogenic shifts during pregnancy, reverting to baseline or a “healthy microbiome” after delivery. These compositional changes during pregnancy may increase susceptibility to harmful oral microorganisms. Once periodontitis establishes, inflammatory cytokines and endotoxins (lipopolysaccharide) are released, leading to elevated systemic levels of neutrophils, CRP, and IL-1, which might amplify inflammatory response, potentially increasing the danger of APOs (17, 35, 75). Nonetheless, Figures 13, 14 indicate that current research on the oral microbiome still predominantly focuses on oral bacterial communities, with the most studies involving *Porphyromonas gingivalis* and *Fusobacterium nucleatum*. *Fusobacterium nucleatum* is closely associated with periodontal disease, and it is not only commonly found in the human oral cavity but also colonizes extra-oral regions such as the placenta and gastrointestinal tract, thereby contributing to APOs and promoting colorectal cancer growth. Given the clinical significance of *F. nucleatum*, there is ongoing research to develop targeted drug therapies aimed at bacterial survival and reproduction. For instance, Bibek et al. (76) combined a xylose-inducible system with a riboswitch, which facilitated the analysis of important genes in *F. nucleatum*, paving the way for potential drug development targeting this bacterium for various clinical applications.

Additionally, the analysis of keywords such as “oral health,” “health,” “adverse pregnancy outcomes,” and “global burden” suggests that the association between oral diseases and systemic health, especially in terms of the burden in different regions and socioeconomic contexts, may become a significant research focus. This is particularly relevant in assessing the impact of PD on developing countries, low-income populations, and maternal-infant health (77). The emergence of “tissue engineering” as a keyword highlights the potential of regenerative techniques, combining biomaterials and stem cell technology, to effectively repair periodontal tissue damage. These advancements offer new therapeutic possibilities for PD regeneration and may help prevent the systemic diseases linked to periodontal conditions (78). This perspective provides a novel approach to reducing the impact of PD on APOs. However, we note that the excessive production of reactive oxygen species (ROS) during biomaterial implantation and cell transplantation may hinder tissue repair, as ROS often lead to severe tissue damage, resulting in cellular injury (79). Moreover, ROS play a complex role during pregnancy, and their balance is critical for maintaining normal pregnancy and embryonic development. Excessive ROS may lead to multiple APOs (80). Currently, many researchers are focusing on the application of ROS scavenging components during tissue regeneration, and the development of such materials could contribute to our understanding of preventing APOs. In the future, we should explore combinations of various interventions in an effort to identify the optimal treatment plan for PD during pregnancy, thereby improving maternal and fetal outcomes.

## 5 Strengths and limitations

### 5.1 Strengths

Compared to traditional literature reviews, which rely on subjective analysis of small sample data and are prone to bias, this study employs bibliometric analysis to provide a statistical overview of previously published literature from 2000 to 2023, examining publication volume, countries, institutions, journals, prolific authors, and keyword co-occurrence. This approach traces the development trajectory of the field of PD and APOs, offering valuable insights for researchers interested in but unfamiliar with the topic. It enables them to quickly grasp the historical research progress, gain a deeper comprehension of the current research landscape, and guide further investigation.

In recent years, there has been a rapid growth in the number of publications concerning PD and APOs; however, few studies have employed bibliometric analysis for systematic evaluation. In 2023, Mayta-Tovalino et al. (81) conducted a bibliometric analysis on the relationship between periodontitis and gestational diabetes but did not expand the scope to include PD and other APOs. In December of the same year, Liu et al. (82) used bibliometric methods to study the development trajectory of PD during pregnancy. However, that study did not explore the impact of PD on pregnancy outcomes. Compared to Mayta-Tovalino's study, this research investigates the connection between PD and multiple APOs, including PB, LBW, and FGR, providing broader applicability. While Liu et al. focused solely on the historical development of PD during pregnancy, this study not only analyzes the historical trends of PD but also systematically examines risk factors and pathways of impact. This multidimensional bibliometric analysis offers more theoretical support for clinical practice and future research. Additionally, by utilizing the most recent literature data (including post-2020 keyword bursts) and incorporating global research trends, this study provides the latest scientific evidence. These innovations in methodology, combined with the expanded scope, depth, and forward-looking perspective, provide a more thorough and systematic examination of the connection between PD and APOs.

### 5.2 Limitations

Consistent with other bibliometric studies, this research has several limitations: (I) Limited Search Scope with a Single Database: This study retrieves data exclusively from the WoSCC database, excluding additional relevant articles from databases such as PubMed and Scopus. This limitation may result in the omission of critical literature on PD and APOs. (II) Citation bias: the analysis may be influenced by citation bias, wherein frequently cited articles dominate due to high citation accumulation or popularity. However, these articles may not comprehensively represent the field or reflect recent developments. Conversely, newly published articles, which may have significant academic value, tend to be undervalued due to their lower citation frequency in the short term. Factors such as publication time, visibility, and journal impact factor contribute to these biases, potentially affecting the outcomes of the analysis. (III) Overemphasis on Quantitative Data: Bibliometric analysis primarily focuses on quantitative metrics, such as citation counts, publication volume, and author collaboration networks, to identify trends and hotspots in academic fields. This quantitative approach may overlook qualitative aspects, including research quality, innovation, methodology, and practical implications. Consequently, significant but less-cited articles may be neglected.

Given these limitations, future studies should incorporate bibliographic databases like PubMed in addition to citation databases such as WoSCC to ensure data comprehensiveness. Tools like VOSviewer can facilitate such multi-database analyses. The retrieved literature should also be systematically screened for content to ensure the quality requirements of the data. Finally, regular updates and dynamic analyses of newly published articles are essential to capture emerging citation trends and maintain the relevance of findings.

## 6 Conclusion

The role of PD in APOs has attracted considerable attention in recent years, as demonstrated by the rising number of related publications. Through bibliometric analysis, this paper offers a thorough overview of research trends, key findings, and the potential impact of PD on shared risks and treatments for APOs. Based on the findings of this study and related research, we identify several important challenges and propose the following recommendations for future research in the PD and APOs field: (I) Risk factors: although the connection between PD and APOs has been widely discussed, the complex interactions between maternal characteristics and overall health still require further clarification. Given the potential shared and modifiable risk factors between PD and APOs, early diagnosis of PD could alert patients to the need for lifestyle changes to minimize the likelihood of negative health outcomes. (II) High-risk populations: screening for risk factors can help identify populations at high risk for APOs. Designing effective interventions for these high-risk groups remains a critical challenge in clinical and public health practice. (III) Oral microbiota and mechanistic research: future studies on the mechanisms by which PD influences APOs should focus more on identifying “key pathogens” and exploring their main virulence factors, as well as investigating the interactions of these pathogens during disease progression, rather than limiting research to the correlation between the severity of periodontitis and Apos' pathological indicators. (IV) Effective interventions: existing studies show uncertainty regarding the effectiveness of clinical interventions for periodontitis in preventing APOs. Future research should emphasize the timing of interventions, the combination of various approaches, and identifying the optimal intervention strategies. (V) International collaboration: with the continued advancement of global research, international collaboration is essential to drive progress in this field. Countries should cooperate in data sharing, standardizing diagnostic and treatment methods, and jointly advancing research on the mechanisms and preventive strategies related to PD and APOs, ultimately improving maternal and neonatal health worldwide.

In conclusion, this study summarizes the knowledge base on PD and APOs, highlights existing research hotspots and emerging trends, offering valuable guidance for future researchers in identifying suitable research directions.

## Data Availability

The original contributions presented in the study are included in the article/Supplementary material, further inquiries can be directed to the corresponding author.

## Supplementary material

Supplementary Table 1 Search strategy.

Supplementary Table 2 Annual publication volume.

Supplementary Table 3 National communications scale.

Supplementary Table 4 Institutional issuance scale.

Supplementary Table 5 Ranking of authors' publications.

Supplementary Table 6 Author co-citation rank.

Supplementary Table 7 Literature co-citation ranking.

Supplementary Table 8 Journal co-citation ranking.

Supplementary Table 9 Keywords Ranking.

Supplementary Table 10 Keywords clustering scale.

Supplementary Table 11 Keywords timeline trends.

Supplementary Table 12 WOSCC literature search string and detailed list of results.

Supplementary Table 13 PubMed literature search string and detailed list of results.

Supplementary Table 14 Sheet 1: The number of adverse pregnancy outcomes (APOs) associated with gingivitis or periodontitis and periodontal disease (including both gingivitis and periodontitis, without significant distinction), respectively. Sheet 2: Articles on the role of each key pathogen in APOs Types of studies and quantitative statistics. Sheet 3: The number of studies on risk (including independent risk of APOs, independent risk of PD, common risk of APOs and PD, and correlation risk of APOs and PD). Sheet 4: Quantitative statistics on ways to prevent APOs. Sheet 5: Classification of articles on the correlation between each type of APO and periodontal disease.

Supplementary Table 15 Quantitative statistics for each study design on PD and APOs [Sheet1: Details of the articles retrieved for each study design; Sheet 2: For specific research design (Animal study, Cross-sectional study, Prospective cohort study, Case-control study, RCT (intervention study), Systematic review), the first three articles with the highest number of citations were collated].
