# Supplementary material for: The association between periodontal disease and adverse pregnancy outcomes: a bibliometric analysis from 2000 to 2023
Source: Front Med (Lausanne). 2025 Jan 21;12:1526406. doi: 10.3389/fmed.2025.1526406 (PMC11790436; doi:10.3389/fmed.2025.1526406)
Supplement: Supplementary Table 1 — Search strategy. [file Table_1.docx]

1.The search string used was TS= ("periodontal disease" OR periodont* OR gingiv* OR "tooth loss" OR "tooth migration" OR "tooth mobility") AND TS= ("Adverse pregnancy outcomes" OR "Miscarriage" OR "Preterm birth " OR "Low birth weight" OR “Congenital anomalies" OR "Stillbirth" OR "Maternal complications" OR "Placental issues" OR "Postpartum complications"), yielding 1,118 records.

2.After adding a time limit from 2000-01-01 to 2023-12-31, the number of records retrieved was reduced to 1,053.

3.After limiting the document types to "Article," "Review Article," or "Early Access," a total of 955 records were retrieved.

4.After limiting the language of the papers to English, a total of 932 articles were retrieved.

Retrieval date：2024-09-22

Database：WOSCC
